# DNA Adenine Methyltransferase (Dam) Overexpression Impairs *Photorhabdus luminescens* Motility and Virulence

**DOI:** 10.3389/fmicb.2017.01671

**Published:** 2017-09-01

**Authors:** Amaury Payelleville, Anne Lanois, Marie Gislard, Emeric Dubois, David Roche, Stéphane Cruveiller, Alain Givaudan, Julien Brillard

**Affiliations:** ^1^Diversité, Génomes Interactions Microorganismes Insectes (DGIMI), Institut National De La Recherche Agronomique, Université de Montpellier Montpellier, France; ^2^MGX-Montpellier GenomiX, Institut de Génomique Fonctionnelle Montpellier, France; ^3^Le Commissariat à l'énergie atomique et aux énergies alternatives (CEA), Genoscope, Université d'Evry, Centre National De La Recherche Scientifique-UMR8030, Université Paris-Saclay Evry, France

**Keywords:** entomopathogenic bacterium, MTase, insect, pathogenicity, flagellar genes, RNA-seq

## Abstract

Dam, the most described bacterial DNA-methyltransferase, is widespread in gamma-proteobacteria. Dam DNA methylation can play a role in various genes expression and is involved in pathogenicity of several bacterial species. The purpose of this study was to determine the role played by the *dam* ortholog identified in the entomopathogenic bacterium *Photorhabdus luminescens*. Complementation assays of an *Escherichia coli dam* mutant showed the restoration of the DNA methylation state of the parental strain. Overexpression of *dam* in *P. luminescens* did not impair growth ability *in vitro*. In contrast, compared to a control strain harboring an empty plasmid, a significant decrease in motility was observed in the *dam*-overexpressing strain. A transcriptome analysis revealed the differential expression of 208 genes between the two strains. In particular, the downregulation of flagellar genes was observed in the *dam*-overexpressing strain. In the closely related bacterium *Xenorhabdus nematophila, dam* overexpression also impaired motility. In addition, the *dam*-overexpressing *P. luminescens* strain showed a delayed virulence compared to that of the control strain after injection in larvae of the lepidopteran *Spodoptera littoralis*. These results reveal that Dam plays a major role during *P. luminescens* insect infection.

## Introduction

Enterobacteria of the genus *Photorhabdus* are highly pathogenic to insects and are also symbiotically associated to nematodes of the family *Heterorhabditidae*. After invasion of the insect host by the nematodes, bacteria are released into the hemocoel of the insect prey where they multiply until septicemia occurs, employing a broad range of virulence factors that kill the insect. The bacterial symbionts contribute to maintain suitable conditions for nematode reproduction. During the final stages of development, the bacteria and the nematode reassociate and subsequently leave the insect carcass in search of a new insect host (Nielsen-LeRoux et al., 2012). As described for many other microbial pathogens, which constantly alternate between their host and the compartment they disperse in Avery (2006), *Photorhabdus* displays phenotypic heterogeneity (Boemare and Akhurst, 1988; Somvanshi et al., 2012). Such phenomena provide diverse phenotypes within a bacterial population, which increases the adaptive potential to rapidly changing environmental conditions (Avery, 2006; Grimbergen et al., 2015). For instance, the production of the *Photorhabdus luminescens* Mad (Maternal adhesion) pilus is controlled by a reversible DNA switch of the promoter of the *mad* operon (Somvanshi et al., 2012). Two forms can co-exist depending on the promoter orientation: the bacteria are in a M-form (for mutualist) when the Mad pilus is produced, because it allows the colonization of the nematode. When the Mad pilus is not produced, the bacteria are in P-form (for pathogenic). In addition, it has been recently demonstrated that the virulence strategy of *P. luminescens* involves the generation of a bacterial subpopulation which causes septicemia in insects by displaying resistance to cationic antimicrobial peptides (Mouammine et al., 2017). Other mechanisms of phenotypic heterogeneity within clonal bacterial cultures exist, such as bistability (Veening et al., 2008). Phenotypic heterogeneity involving a positive-feedback loop regulation of a transcriptional regulator has been reported in the closely related genus *Xenorhabdus*, where it controls the expression of motility and virulence determinants (Jubelin et al., 2013). DNA methylation is another process that has also been described as responsible for bacterial phenotypic heterogeneity (Casadesus and Low, 2013), but this has not been investigated in *Photorhabdus*.

Numerous DNA methyltransferases (MTases) are a component of restriction-modification (RE) systems that protect the bacterial cell from invasion by bacteriophages DNA (Marinus and Løbner-Olesen, 2014). Other MTases are not linked to restriction endonuclease and are classified as “orphan” MTases (Casadesus, 2016). The best characterized orphan MTase in bacteria is Dam (DNA adenine methyltransferase), originally identified in *Escherichia coli* and widespread among gamma-proteobacteria (Lobner-Olesen et al., 2005). Dam MTase transfers a methyl group to an adenosine localized in sites 5′-GATC-3′ of the DNA. After DNA replication, the newly synthetized DNA strand is unmethylated and the GATC sites are therefore transiently hemimethylated (Wion and Casadesus, 2006; Marinus and Casadesus, 2009) until the action of a Dam protein. The Dam enzyme has key roles in bacterial genome maintenance, and *E. coli dam* mutants have defects in many important physiological processes such as DNA replication initiation, chromosome partitioning, nucleoid structure, and mismatch repair (Lobner-Olesen et al., 2005).

Changes in DNA methylation can also alter the affinity of regulatory proteins to their target DNA binding sequences (Hale et al., 1994; Tavazoie and Church, 1998). Conversely, DNA-binding proteins can represent a hindrance to MTase for reaching specific DNA sequences, and therefore can inhibit DNA methylation. Both mechanisms may lead to alterations in gene expression. Thus, DNA-methylation can play major roles in transcriptional regulation, including genes involved in bacterial virulence (Heusipp et al., 2007). An illustrative example is the Pap (pyelonephritis-associated pili) pilus phase variation of uropathogenic *E. coli*. The *pap* operon transcription depends on the binding of the transcriptional repressor Lrp. Such binding depends on the methylation state (on several GATC sites) of the *pap* promoter because of a better Lrp affinity for non-methylated DNA (Blyn et al., 1990; Braaten et al., 1994). The regulation of gene that mediates adhesion to uroepithelial cells is therefore governed by DNA methylation state. In *E. coli*, Dam regulates transcription of several other pili operons (van der Woude and Low, 1994), and expression of a major outer-membrane protein (Ag43) (Henderson and Owen, 1999).

In several bacterial species that possess a *dam* gene, Dam has been described as an important virulence gene regulator. Mutants of *Salmonella* Typhimurium lacking the Dam enzyme are avirulent in mice (Garcia-Del Portillo et al., 1999; Heithoff et al., 1999). The impact of Dam inactivation on bacterial virulence has also been reported in *Haemophilus influenzae* (Watson et al., 2004), *Klebsiella* (Mehling et al., 2007), *Actinobacillus* (Wu et al., 2006) and *Yersinia pestis* (Robinson et al., 2005). In *Yersinia pseudotuberculosis, Vibrio cholera*, and *Aeromonas hydrophila*, inactivation of the *dam* gene was shown to be a lethal mutation (Julio et al., 2001; Erova et al., 2006b; Demarre and Chattoraj, 2010). However, plasmid-mediated overexpression of the *dam* gene in *Y. pseudotuberculosis* resulted in a virulence decrease in mice compared to wild-type (Julio et al., 2002) and in a defect in colonization of *V. cholerae* in a suckling mouse model compared to wild-type (Julio et al., 2001). Similarly, *dam*-overexpressing strains of *Salmonella, Pasteurella multocida*, or *A. hydrophila* were also highly attenuated in mice (Heithoff et al., 1999; Chen et al., 2003; Erova et al., 2006b).

Phenotypes associated with an alteration of the DNA methylation state have mostly been described in mammalian pathogens, but have not yet been reported in an insect-pathogenic bacterium. The gene plu0087 of the *P. luminescens* TT01 genome is annotated “DNA adenine methylase (Deoxyadenosyl-methyltransferase)” (Duchaud et al., 2003) and displays 70.7% of identity and 86.3% of similarity with the *E. coli dam* gene. The purpose of this study was to determine the role of deregulating the *dam* orthologous gene found in *P. luminescens* TT01. In this work, we confirmed the N^6^-Adenine methyltransferase function of the *dam* gene product. Investigation of several phenotypes of a *P. luminescens dam*-overexpressing strain revealed a major role for Dam in motility as well as during virulence in the insect.

## Materials and methods

### Strains and growth conditions

The bacterial strains and plasmids used in this study are listed in Table 1. *E. coli, P. luminescens* and *Xenorhabdus nematophila* cells were routinely grown in Luria broth (LB) medium with a 180 rpm agitation at 37 and 28°C, respectively. As required, antibiotic concentrations used for bacterial selection were gentamycin at 15 μg mL^−1^, rifampicin at 100 μg mL^−1^ and kanamycin at 20 μg mL^−1^. IPTG was added at 0.2 mM when required.

**Table 1:** Strains and plasmids used in this work.

**Strain or plasmid**	**Relevant genotype and characteristics^a^**	**Reference or source**
**STRAINS**		
*Photorhabdus luminescens* TT01	Wild type	Duchaud et al., 2003
*Escherichia coli* XL1 blue MRF'	*Δ(mcrA)183 Δ(mcrCB-hsdSMR-mrr)173 endA1 supE44 thi-1 recA1 gyrA96 relA1 lac [F′ proAB lacIqZΔM15 Tn10 (Tetr)]*	Agilent Technologies
*E. coli WM3064*	*thrB1004 pro thi rpsl hsdS lacZΔM15 RP4-1360Δ(araBAD)567 ΔdapA1341::[erm pir (wt)]*	Paulick et al., 2009
*E. coli* MG1655	Wild type	Lobner-Olesen and von Freiesleben, 1996
*E. coli Dam::16KM*	MG1655 *dam16*::Km^r^	Lobner-Olesen and von Freiesleben, 1996
*Xenorhabdus nematophila* F1	Wild type	Lanois et al., 2013
*Micrococcus luteus*	Wild type	Pasteur Institute Culture collection, Paris, France
**PLASMIDS**		
pBBR1MCS-5	Cloning vector, Gm^r^	Kovach et al., 1995
P_tet_-MCS	Cloning vector, Km^r^	Jubelin et al., 2013
P_tet_-MCS-Dam	853 pb PCR fragment (*dam* gene) inserted between KpnI and BamHI site of P_tet_-MCS plasmid	This study
pBB-Dam	864 pb fragment (RBS and *dam* gene) isolated from P_tet_-MCS-Dam and inserted between EcoRI and BamHI site of pBBR1MCS-5 plasmid	This study
pJQ200KS	Mobilizable vector, Gm^r^	Quandt and Hynes, 1993
pJQ-Δdam	Region overlapping the *dam* gene disrupted by a Cm^r^ cassette and inserted between PstI and XbaI site of pJQ200KS plasmid	This study

a *Km, kanamycin; Gm, gentamicin; Cm, chloramphenicol*.

### *In silico* analysis

Primer sequence was designed using the Primer3 software (Untergasser et al., 2012). The REBASE database (Roberts et al., 2015) was used to identify a putative Dam methyltransferase, M.PluTDamP (REBASE Enzyme Number 7410), in the *P. luminescens* TT01 genome. Alignments between Dam from various organism were performed using the Multalin tool (Corpet et al., 1998).

### Nucleic acid manipulations

The extraction of plasmid DNA from *E. coli* was performed using the GenElute™HP Plasmid® miniprep purification kit as recommended by the manufacturer (Sigma). Chromosomal DNA was extracted from bacterial cells using the QIAamp DNA Mini kit (Qiagen). Restriction enzymes and T4 DNA ligase were used as recommended by the manufacturer (New England Biolabs and Promega, respectively). Oligonucleotide primers were synthesized by Eurogentec (Seraing, Belgium) and are listed in Table S1. PCR was performed in a T100 thermal cycler (Biorad) using the iProof high-fidelity DNA polymerase (Biorad). Amplified DNA fragments were purified using a PCR purification kit (Roche) and separated on 0.7% agarose gels after digestion as previously described (Brillard and Lereclus, 2007). Digested DNA fragments were extracted from agarose gels with a centrifugal filter device (DNA gel extraction kit, Millipore, Molsheim, France). All constructions were confirmed by DNA sequencing (Eurofins Genomics).

Cloning the *P. luminescens dam* gene was performed as follows. The plu0087 gene was PCR amplified using two primers mapping immediately upstream and downstream (Cp-plu087-F and Cp-plu087-R, respectively, Table S1) the 804 bp ORF (open reading frame), using the following cycling conditions: 98°C, 10 s; 56°C, 30 s; 72°C, 30 s for 35 cycles. The 853 bp-long amplified DNA fragment was then digested according to the endonuclease sites introduced in the primers (KpnI and BamHI). Because the *P. luminescens dam* gene has no clear ribosome binding site (RBS), the generated DNA fragment was first inserted immediately downstream of the RBS already present in the plasmid P_tet_-MCS (Jubelin et al., 2013), to create the P_tet_-MCS-Dam (Table 1). A 864 bp-long fragment corresponding to this insert together with the RBS was then isolated from the P_tet_-MCS-Dam plasmid by EcoRI and BamHI endonucleases, and was inserted between the corresponding sites of the low-copy plasmid pBBR1MCS-5 (Kovach et al., 1995) downstream of the P_*lac*_ promoter. The recombinant plasmid (pBB-dam) was introduced in *E. coli* strains by electroporation, or transferred in *P. luminescens* and *X. nematophila* by conjugative mating as previously described (Givaudan and Lanois, 2000). Transconjugants harboring the pBBR1MCS-5 empty plasmid were used as a control.

Attempts to construct a *dam* mutant were performed as follows. Briefly, DNA fragments of the plu0087 upstream (540 pb) and downstream (569 bp) regions were PCR-amplified using the primer pairs upF-plu0087/upR-plu0087 and dnF-plu0087/dnR-plu0087, respectively (Table S1). PCR products were digested with PstI/BamHI and BamHI/XbaI using the primer-incorporated restriction sites (Table S1). In parallel, the Ω interposon harboring a Cm^r^ cassette was digested with BamHI, as previously described (Brillard et al., 2002). The three digested DNA fragments were purified, ligated in PstI/XbaI-digested pJQ200KS (Table 1), and introduced by electroporation in *E. coli* XL1. The resulting pJQ-Δdam plasmid was transferred in *P. luminescens* by conjugative mating. Four independent transconjugants clones were then subjected to allelic exchange in LB at 28°C, following the protocol routinely used in the laboratory (Brillard et al., 2002; Derzelle et al., 2004b; Brugirard-Ricaud et al., 2005). Because several attempts were unsuccessful, the transconjugants were additionally subjected to allelic exchange in a M9 minimal medium instead of LB, or incubated at room temperature or at 15°C instead of 28°C.

### DNA methylation state analysis in *E. coli dam* mutant expressing the *P. luminescens dam* gene

The methylation state of GATC sites was assessed in an *E. coli dam* mutant strain Dam 16::KM (Lobner-Olesen and von Freiesleben, 1996) harboring pBB-Dam or the pBBR1MCS-5 empty plasmid, in order to determine the *P. luminescens dam* functionality in *E. coli*. These strains were grown in LB supplemented with gentamycin (to maintain the plasmid) and IPTG (Isopropyl β-D-1-thiogalactopyranoside) to allow the activation of the P_*lac*_ promoter controlling the expression of the *dam* gene. Fifty nanogram of plasmid DNA extracted from these strains were then digested during 2 h at 37°C by MboI (which digests only non-methylated GATC sites) or DpnI (which digests only methylated GATC sites) and DNA digestions were analyzed after electrophoresis on a 1% agarose gel.

### Phenotype analysis of *P. luminescens*

Bromothymol blue adsorption was determined after growth on NBTA (nutrient agar supplemented with 25 mg of bromothymol blue and 40 mg of triphenyltetrazolium chloride per liter). It allows the identification of variant forms (Boemare and Akhurst, 1988). Antibiotic production was assessed by measuring antibacterial activity against *Micrococcus luteus* (Table 1). Hemolysis was determined by the observation of a clearing surrounding bacteria grown on standard sheep blood agar plates as previously described (Brillard et al., 2001). Bioluminescence production, lipase activity on Tween 20, 40, 60, 80, and 85 were also assessed as previously described (Boemare and Akhurst, 1988).

For motility assays, agar plates were prepared with LB broth supplemented with 0.35% agar and inoculated using 5 μL of cells grown in exponential phase (OD_540 nm_ = 0.8), as previously described (Givaudan et al., 1995). The diameter of the halo size of swimming motility was measured 24 h and 30 h after incubation. Data from 3 independent experiments (with 10 plates used in each condition) were analyzed using Wilcoxon test.

Growth of *P. luminescens* was monitored with a TECAN automated turbidimetric system (Infinite M200 TECAN®). Estimation of maximum specific growth rate (μ_max_) was performed on 4 independent biological replicates for each strain, using serial dilution of the inoculum as previously described (Augustin et al., 1999).

The *P. luminescens* biofilm formation was determined as follows. Five milliliters of LB medium in glass tubes were inoculated at 10% with an overnight culture and incubated for 12 days at 28°C in static conditions. The tubes were then rinsed with PBS before the addition of 7 mL of Crystal violet solution at 0.01% (in PBS) to stain the biofilms during 15 min. Biofilms were rinsed with PBS and then dissolved 3 h in 7 mL ethanol. The OD_570 nm_ measurement allowed the quantification of the biofilm-associated crystal violet. Data from 3 independent experiments with replicates (totalizing 17 tubes for each strain) were analyzed using Wilcoxon test.

The *P. luminescens* spontaneous mutation rate was assessed by quantifying the emergence of rifampicin-resistant CFUs as follows. *P. luminescens* was grown overnight in 100 mL of LB medium supplemented with gentamycin (for plasmid maintenance) before plating on LB and LB with rifampicin. The mutation rate was calculated as the rifampicin-resistant population divided by the total population. Data from 3 independent experiments were compared using the Student *t*-test.

### Plasmid curing of *P. luminescens* strains

Plasmid curing was performed as follows. For each *P. luminescens* transconjugant strain, a fresh colony was used to inoculate 5 mL LB and incubated overnight with shaking in the absence of antibiotic pressure. These cultures were used to inoculate 100 mL fresh LB at an OD_540 nm_ = 0.05, and incubated with shaking until OD_540 nm_ = 0.8 was reached. These cultures were then diluted and spread on LB agar-plates prior incubation until CFU were visible. For each strain, 50 CFU were then streaked on LB Gm and LB without antibiotic in parallel to check for the plasmid stability. The loss of the pBB-Dam plasmid, as well as that of the pBBR1MCS-5, was observed for all the 50 tested CFU. Four Gm^S^ clones from each strain were then tested for their motility ability after inoculation on low-agar plates as described above. Finally, one clone from each strain was tested for insect virulence.

### Insect virulence assay

The virulence-related properties of *dam*-overexpression were assessed by comparing the killing effect of *P. luminescens* transconjugants harboring either the pBB-Dam or the pBBR1MCS-5 empty plasmid during infection in the common cutworm *Spodoptera littoralis* as previously described (Brillard et al., 2002). Four to five independent pathogenicity assays were performed for each bacterial strain. Briefly, 20 μL of exponentially growing bacteria (DO_540 nm_ = 0.3) diluted in LB, corresponding to about 1 × 10^4^ CFU (1.4 × 10^4^, mean value of 4 experiments or 1.2 × 10^4^, mean value of 5 experiments, for *P. luminescens* harboring pBBR1MCS-5 or pBB-Dam, respectively), were injected into the hemolymph of 20 fifth-instar larvae of *S. littoralis* reared on an artificial diet. Insect larvae were then individually incubated at 23°C. Altogether, the survival rate of 80–100 larvae for each bacterial strain were analyzed. The CFU of bacteria were determined by plating dilutions on LB agar. Insect death was monitored over time for up to 60 h. The time for killing 50% of the insect larvae (LT_50_) was calculated. Statistical analysis (Wilcoxon test) was performed as previously described (Givaudan and Lanois, 2000; Brillard et al., 2002) using SPSS version 14.0 (SPSS, Inc., Chicago, IL) to compare the mortality state.

### RNA preparation

Total RNA extraction was performed on cells harvested at OD_540 nm_ = 0.5, from nine independent cultures for each strain, using RNeasy miniprep Kit (Qiagen), according to the manufacturer's instructions. An additional incubation step with DNase I (Qiagen) was performed. The quantity and quality of RNA were assessed with an Agilent 2100 Bioanalyzer with the RNA 6000 Nano LabChip kit. Lack of DNA contamination was controlled by carrying out a PCR on each RNA preparation.

### RNA sequencing

The RNA-sequencing was performed as previously described (Mouammine et al., 2017) with the following changes. Equal amounts of total RNA from three independent samples per strain were pooled together to generate one final biological RNA sample per strain. Thus, from the initial nine independent RNA samples per strain, three final RNA samples were generated for each strain and subsequently treated as follows prior sequencing. Ribo-Zero rRNA Removal Kit Bacteria (illumina, San Diego, CA) was used to remove ribosomal RNA from 4 μg of total RNA. For each sample, 100 ng of rRNA-depleted RNA was used to construct sequencing libraries using Illumina's TruSeq Stranded mRNA Sample Prep Kit (Low throughput). The mRNA was chemically fragmented. The first cDNA strand was generated by reverse transcription with random hexamer primers, SuperScript IV Reverse Transcriptase (Life Technologies), Actinomycine D. The Second strand cDNA was synthesized by replacing dTTP with dUTP. A single “A” nucleotide was added to the 3′ end and ligation was carried out with Illumina's indexed adapters. After 15 cycles of PCR, libraries were validated on a Fragment Analyzer (AATI, Ankeny, IA) and quantified with a KAPA qPCR kit. On a sequencing lane of a flowcell V4, nine libraries were pooled in equal proportions, denatured with NaOH and diluted to 8 pM before clustering. Clustering and 50 nt single read sequencing were performed according to the manufacturer's instructions. Cluster formation, primer hybridisation and single end-read 50 cycles sequencing were performed on cBot and HiSeq2500 (Illumina, San Diego, CA), respectively. Image analyses and basecalling were performed using the Illumina HiSeq Control Software and Real-Time Analysis component. Demultiplexing was performed using Illumina's conversion software (bcl2fastq 2.17). The quality of the data was assessed using FastQC from the Babraham Institute and the Illumina software SAV (Sequencing Analysis Viewer). Potential contaminants were investigated with the FastQ Screen software from the Babraham Institute.

### RNA-seq analysis

High-throughput transcriptomic sequencing data were processed with a bioinformatic pipeline implemented at the Microscope platform (Vallenet et al., 2013). The reads were mapped onto the *P. luminescens subsp. laumondi* TT01 genome sequence (EMBL accession number: BX470251) with BWA software (v. 0.7.4) (Li and Durbin, 2009). We then used SAMtools (v.0.1.12) (Lister et al., 2009) to lower the false-positive discovery rate and to extract reliable alignments from BAM-formatted files. The number of reads matching each genomic object harbored by the reference genome was then calculated with the Bioconductor-GenomicFeatures package (Lawrence et al., 2013). For reads matching several genomic objects, the count number was weighted so as to keep the total number of reads constant. Finally, we used the Bioconductor-DESeq package (Anders and Huber, 2010) with default parameters to analyze raw count data, to normalize the samples to the reliable reads and to evaluate differential expression between conditions, as previously described (Jubelin et al., 2013). Between 14 and 19 million Illumina sequences (50-base reads) were obtained for each sample and between 80 and 93% of high-quality sequences mapped to at least one site in the reference genome. The complete dataset from this study has been deposited in NCBI's Gene Expression Omnibus (GEO) database, under accession number GSE100650.

### RT-qPCR analysis

For the validation of RNA-seq data, quantitative reverse transcription-PCR (RT-qPCR) were carried out as previously described (Mouammine et al., 2017). Briefly, RNA samples from 9 biological replicates for each strain were used for cDNA synthesis. The SuperScript II reverse transcriptase (Invitrogen) was used on 1 μg of total RNA with random hexamers (100 ng/μl; Roche Diagnostics). qPCR analyses were performed using SYBR green Master kit (Roche Diagnostics) with 1 μl of cDNA and specific gene primers at 1 μM (Table S1). The reactions were performed in triplicate at 95°C for 10clones from each strain min, followed by 45 cycles at 95°C for 5 s, 61°C for 10 s, and 72°C for 15 s and monitored in the LightCycler 480 system (Roche). Melting curves were analyzed and always contained a single peak. The data analyzed with the REST software 2009 (Pfaffl et al., 2002) using the pairwise fixed randomization test with 2,000 permutations are presented as a ratio with respect to the reference housekeeping gene *gyrB*, as previously described (Jubelin et al., 2013).

## Results

### Conservation of major amino acids in *P. luminescens* dam protein

Functional characterization of Dam proteins has been described in several organisms, and allowed the identification of several amino-acids essential for the Dam function (Yang et al., 2003; Erova et al., 2006a; Horton et al., 2006). Comparing these protein sequences with that of M.PluTDamP by a multiple alignment revealed that all major amino acids described in these organisms were conserved in *P. luminescens* Dam protein (Figure S1). They were also conserved in a protein encoded by a *dam* ortholog (XNC3v2_1950011) (83.3% of identity and 92.6% of similarity with M.PluTDamP) found in the closely related bacterium *X. nematophila* (Lanois et al., 2013). These findings strongly suggest that M.PluTDamP plays a role in DNA adenine methylation, as described for other Dam proteins.

### Complementation by M.PluTDamp of an *E. coli dam* mutant

**Figure 1:**
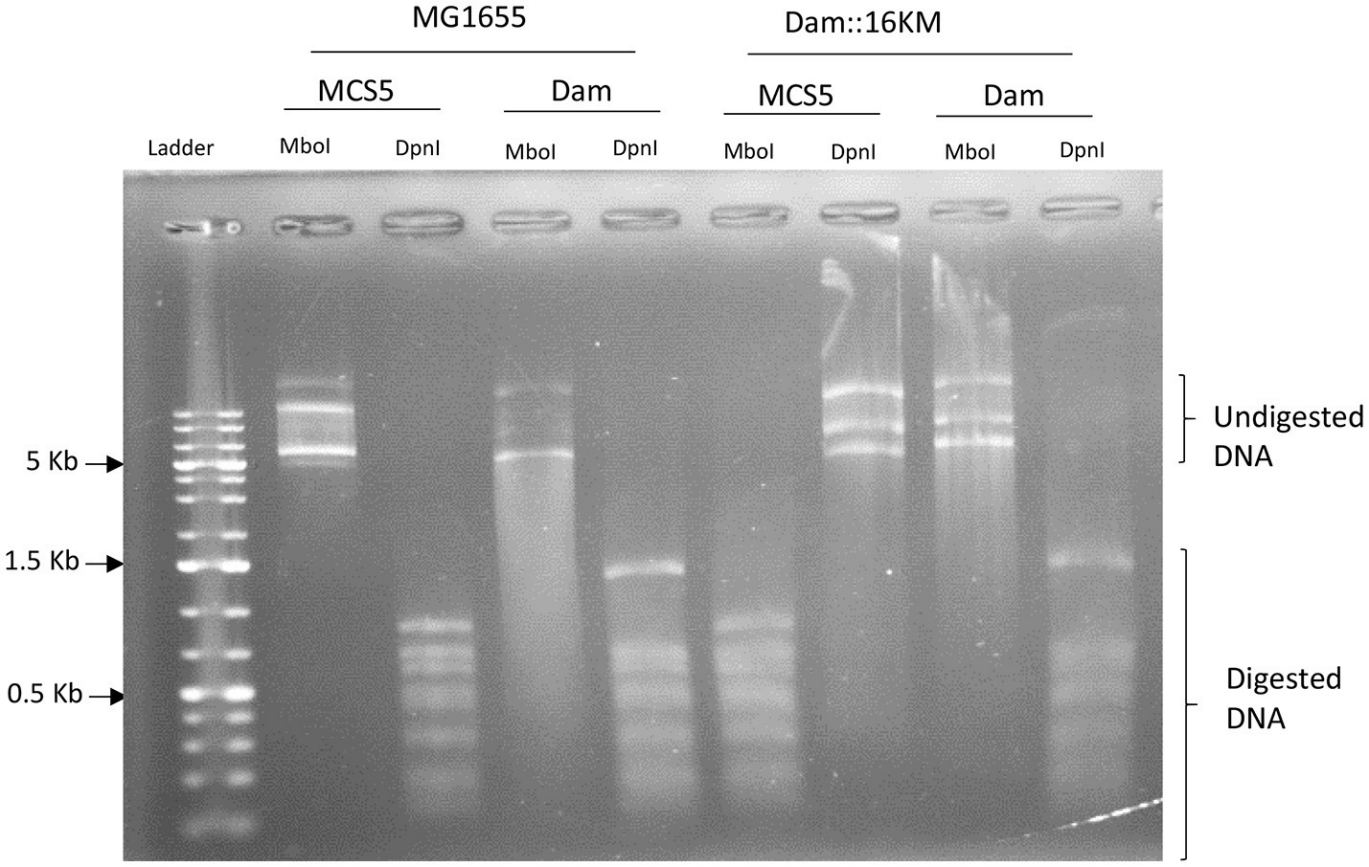
Differential plasmid DNA digestion from *E. coli*. An *E. coli dam* mutant (Dam::16KM) or its parental strain (MG1655) were complemented with either a plasmid harboring the *P. luminescens dam* gene (Dam) or the pBBR1-MCS5 empty vector (MCS5). Plasmid DNA was extracted and digested by MboI (active on unmethylated 5′-GATC-3′ sites) or DpnI (active on 5′-GmeATC-3′ sites). pBBR1-MCS5 (4,768 bp) has 17 GATC sites and pBB-Dam (5,618 bp) has 19 GATC sites.

In order to confirm the *P. luminescens* Dam function, the plu0087 gene was cloned and introduced in an *E. coli dam* mutant or in its *E. coli* parental strain. The pBBR1MCS-5 empty plasmid was used as a control. The recombinant strains harboring pBB-Dam or pBBR1MCS-5 were assessed for their ability to methylate GATC sites. Plasmid DNA extracted from these strains were digested by enzymes sensitive to DNA methylation. Results presented in Figure 1 show that the DNA extracted from the *E. coli* MG1655 strain harboring either the pBB-Dam or the empty plasmid was digested by DpnI, but not by MboI, as expected, indicating that the GATC sites of the DNA are methylated in this strain, whatever the plasmid introduced. However, the DNA extracted from the *E. coli dam* mutant harboring the control empty plasmid was digested by MboI, but not by DpnI, confirming that the GATC sites are not methylated in this mutant strain. In contrast, the DNA extracted from the *E. coli dam* mutant harboring the pBB-Dam was digested by DpnI, but not by MboI, revealing a methylation on GATC sites. This indicates that the *P. luminescens dam* gene was able to complement the *E. coli dam* mutant, and therefore confirms that it is a genuine *dam* ortholog, with M.PluTDamP being able to methylate adenine on GATC sites of DNA.

### *dam* overexpression in *P. luminescens* does not alter growth nor several major phenotypes

Construction of a *P. luminescens dam*-mutant failed despite several attempts. The role of *P. luminescens dam* gene was therefore investigated by using a strain overexpressing *dam*. In *P. luminescens*, genes placed under the control of the P_*lac*_ promoter are constitutively expressed (Lanois et al., 2011; Mouammine et al., 2014). Therefore, the additional copy of the *dam* gene caused by the presence of pBB-Dam plasmid, together with constitutive expression of the strong P_*lac*_ promoter are supposed to induce a *dam* overexpression in *P. luminescens*. This postulate was confirmed by quantification of mRNA corresponding to the *dam* gene. RT-qPCR experiments showed an average of 23.1-fold induction of expression of *dam* in *P. luminescens* harboring pBB-Dam when compared to the control strain (ie, harboring a pBBR1MCS-5 empty plasmid).

**Figure 2:**
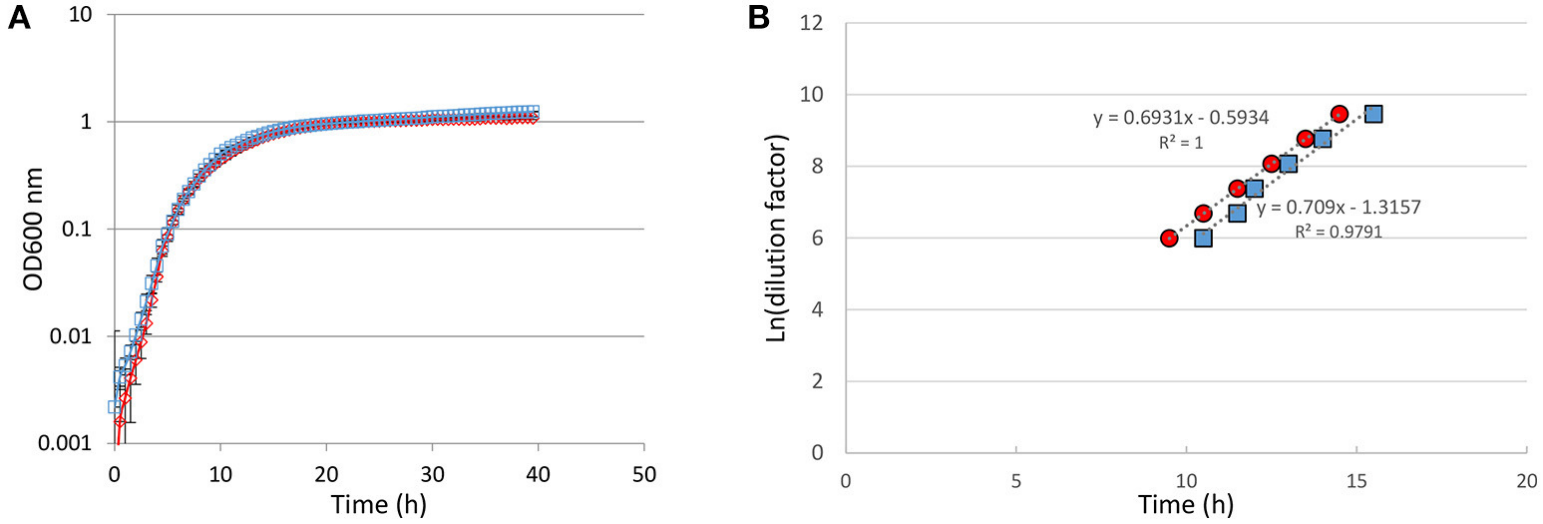
Growth comparison between *P. luminescens* overexpressing *dam* gene (red) and control harboring an empty plasmid (blue). **(A)** Growth curves in LB of the *P. luminescens* Dam-overexpressing strain and control strain (inoculated at 1.61 × 10^5^ and 1.86 × 10^5^ CFU/ml, respectively). Mean values ± SDs of at least 3 independent biological replicates for each strain are shown. **(B)** Two-fold serial dilutions of cultures containing 1.25 × 10^4^ CFU/ml for each strain have been performed in LB. Growth was quantified by absorbance at 600 nm and measured every 30 min. Four independent biological replicates for each strain were used. The time of growth detection was defined as an increase of 0.1 unit of absorbance at 600 nm, and recorded for each dilution. The Ln of the dilutions as a function of the time for growth detection is indicated. Similar slopes indicate similar growth rates (see Methods section for details). Differences were not significant (*p* = 0.92, Student *t*-test).

Considering that this *dam* overexpression may modify *P. luminescens* physiology, growth of both strains was monitored with an automated turbidimetric system, and the maximum specific growth rate (μ_max_) was estimated (Figure 2). The growth curves of both strains overlapped with the same shape: their slope were similar during the exponential phase and they reached the same maximum OD during stationary phase. No lag-phase was observed. Moreover, the calculated μ_max_ were not different: 0.647 h^−1^ for *P. luminescens* harboring pBB-Dam vs. 0.636 h^−1^ for the control strain (*p* = 0.92, Student *t*-test). Several phenotypes were also assessed to compare the *dam*-overexpressing strain to the control strain. No significant difference was observed between *P. luminescens* harboring pBB-Dam when compared to the control *P. luminescens* strain for bromothymol blue adsorption on NBTA, bioluminescence production, antibiotic production, hemolysis, lipase activities and mutation rate (Table 2). Altogether, these findings indicate that many *P. luminescens* phenotypes are not altered by *dam* overexpression.

**Table 2:** Phenotypes of *P. luminescens* TT01 transconjugants, overexpressing *dam* gene (pBB-dam) and control (pBBR1MCS-5).

		**Tested phenotypes**^**a**^										
**Strain**	**Btb adsorption^b^**	**Bioluminescence^c^**	**Antibiotic production^d^**	**Sheep blood hemolysis^e^**	**Motility^f^**	**Lipolysis of**^**g**^					**Growth rate (h^−1^)^h^**	**Mutation rate^i^**
						**Tween 20**	**Tween 40**	**Tween 60**	**Tween 80**	**Tween 85**		
TT01 WT	G	+	+	−	++	+	+	+	+	−	ND	ND
TT01+pBBR1MCS-5	G	+	+	−	++	+	+	+	+	−	0.636	4.80 × 10^−8^
TT01+pBB-dam	G	+	+	−	+	+	+	+	+	−	0.647	3.84 × 10^−8^

a *All plates were incubated for 2 days at 28°C before assays were interpreted, unless otherwise indicated. Routinely tested phenotypes on the WT strain are indicated for comparison*.

b *Btb, bromothymol blue; G, green-blue colonies on NBTA medium*.

c *+, Luminescence detected by visual observation in a dark room*.

d *+, Halo size (>25 mm) of growth inhibition of Micrococcus luteus*.

e -, No halo of hemolysis detected

f *++, Large spreading area (halo size >20 mm); +, reduced spreading area (halo size < 20 mm) after 30 h of incubation*.

g +, Halo of precipitation; −, no halo of precipitation

h *Growth rate was estimated on 4 independent biological replicates, using serial dilution of the inoculum as described in the method section. Differences were not significant (p = 0.92, Student t-test). ND, not done*.

i *Spontaneous mutation rate per CFU was assessed by quantifying the number of rifampicin-resistant CFU arising in the total population after overnight growth in liquid medium. Presented values are the mean of 3 independent experiments. Differences were not significant (p = 0.73, Student t-test)*.

### *dam* overexpression impairs the bacterial motility

**Figure 3:**
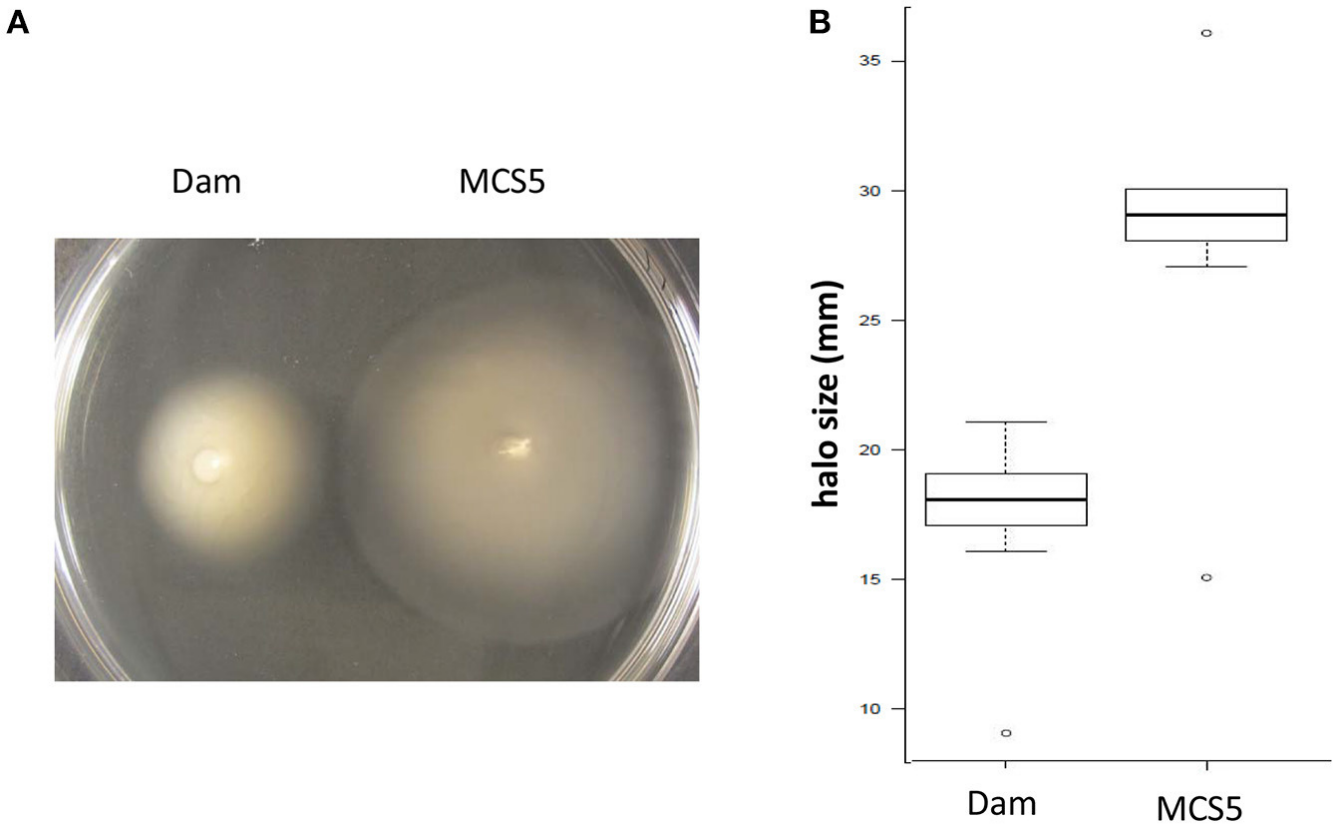
Swimming motility of *P. luminescens* overexpressing *dam* gene (Dam) and control (MCS5). **(A)** Swimming halos were observed on low agar LB medium inoculated by 5 μL of exponentially growing cells. **(B)** Boxplots of the diameter of the halo size of motility of each strain measured after 30 h of incubation (see Methods section for details). Difference between the two strains is significant (*p* < 0.001, Wilcoxon test).

The halo size of motility, assessed on low agar LB medium, was much smaller in *P. luminescens* harboring pBB-Dam when compared to that of the control strain (Figure 3). Data revealed that for *P. luminescens* harboring pBB-Dam, the median halo size after 30 h of incubation was 59.3% that of the control strain (17.5 vs. 29.5 mm, respectively). This difference was significant as early as 24 h after inoculation (*p* < 0.01, Wilcoxon test), and highly significant after 30 h of incubation (*p* < 0.001, Wilcoxon test). This indicates that motility is significantly reduced but not abolished in the *dam*-overexpressing *P. luminescens* strain.

Because introducing the pBB-Dam plasmid led to an impaired motility, we checked if the loss of the plasmid would restore the WT phenotype. After growth in a liquid medium followed by growth on agar plates, both in the absence of antibiotic pressure, the loss of the pBB-Dam plasmid, as well as that of the pBBR1MCS-5, was observed for all the 50 tested CFU of *P. luminescens* (not shown). Four Gm-sensitive CFU originating from each strain were then tested for their motility ability after inoculation on low-agar plates, as described above. No significant difference in the halo size of motility was observed between the cured strains originating either form the *dam*-overexpressing *P. luminescens* strain or from the control strain, after 30 h of incubation (*p* = 0.44, Wilcoxon test). These results revealed that motility was fully restored in pBB-Dam cured cells, confirming that *dam*-overexpression causes an impaired motility in *P. luminescens*.

We wondered if *dam* overexpression in another species closely related to *P. luminescens* would also cause an impaired motility. We therefore introduced the pBB-Dam plasmid, or the control empty plasmid, in *X. nematophila* and checked the motility on a low agar medium. Results from 5 independent experiments showed that the median halo size after 30 h of incubation was 13 mm for *X. nematophila* harboring pBB-Dam vs. 19 mm for the control strain. This difference was significant (*p* = 0.001, Wilcoxon test) indicating that swimming motility is reduced, but not abolished, in the *dam*-overexpressing *X. nematophila* strain, similarly as it was observed for *P. luminescens*.

### *dam* overexpression increases the biofilm formation ability

**Figure 4:**
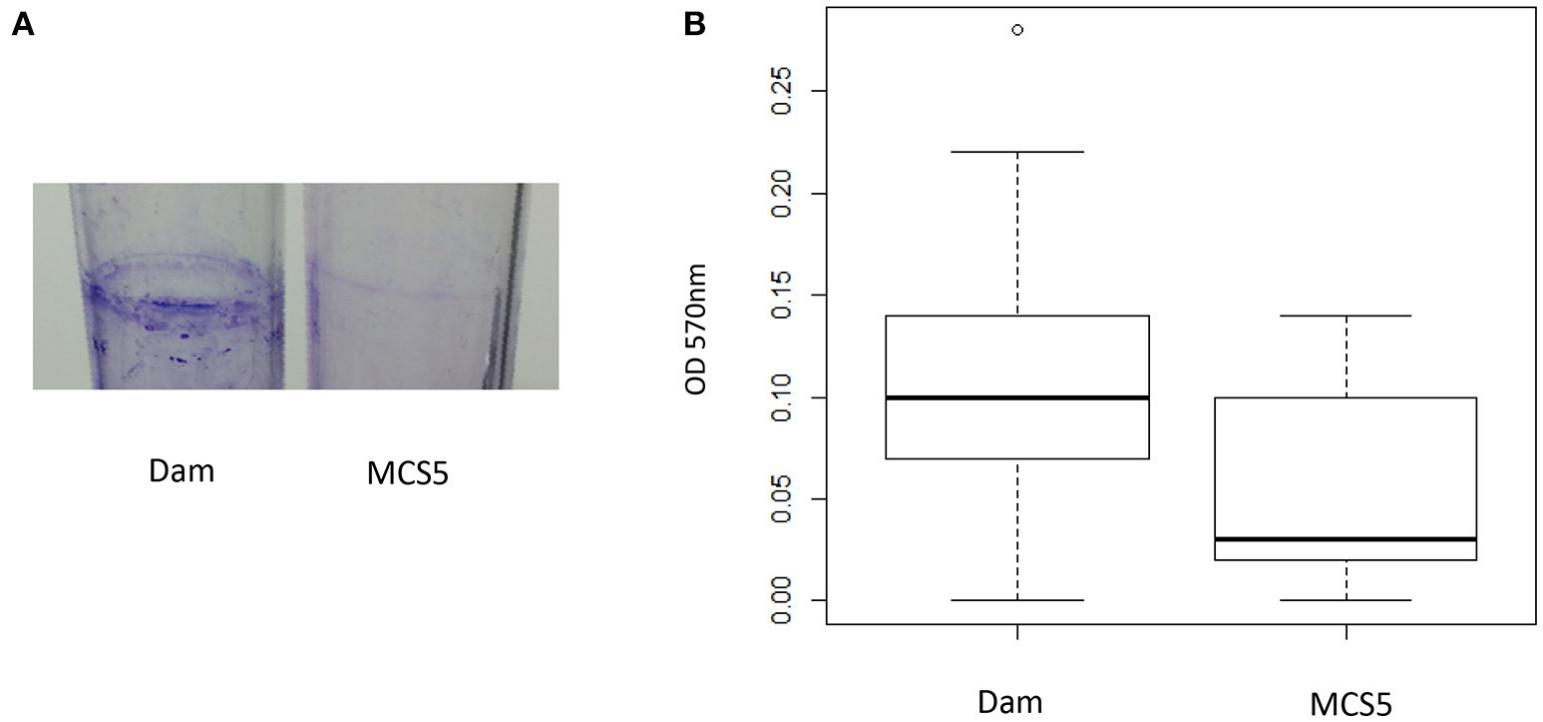
Biofilm formation ability of *P. luminescens* overexpressing *dam* gene (Dam) and control (MCS5). **(A)** Biofilms formed in a glass tube after 12 days of incubation in LB medium were stained with crystal-violet. **(B)** Boxplots of the biofilm-associated crystal violet measured at OD_570 nm_ (see Methods section for details). Difference between the two strains is significant (*p* < 0.01, Wilcoxon test).

Determination of the biofilms formed in glass tubes by the *P. luminescens* strain overexpressing *dam* and the control strain was analyzed by a crystal violet staining method (Figure 4). The results revealed a significant increase of biofilm-associated crystal violet measured for the *P. luminescens* overexpressing *dam* strain (*p* = 0.003, Wilcoxon test), suggesting an increase in adhesion properties for this strain.

### Effect of *dam* overexpression on *P. luminescens* insect virulence

**Figure 5:**
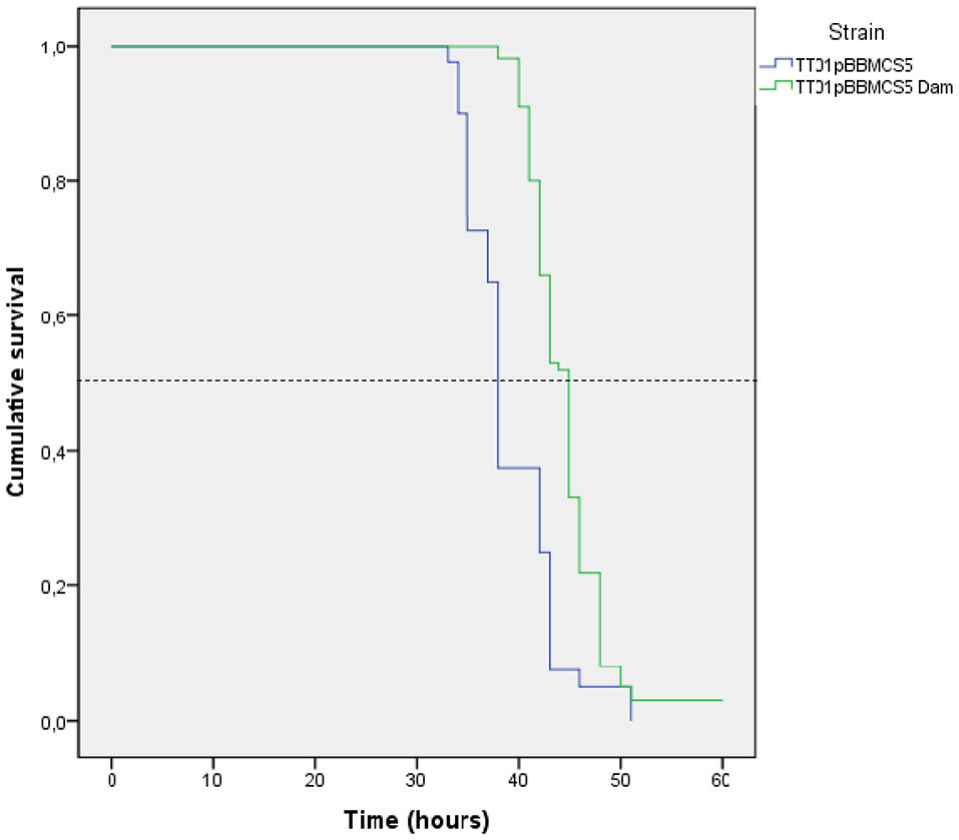
Infection of *Spodoptera littoralis* larvae by *P. luminescens* overexpressing *dam* and the control strain. Proportion of survival of *S. littoralis* after injection of 10^4^ CFU of *P. luminescens* overexpressing *dam* (TT01 + pBB-Dam, green) or carrying the vector control (TT01 + pBBR1-MCS5, blue). Graph represents the results from 4 to 5 independent experiments (with 20 insect larvae per experiment). The survival of 50% of the infested larvae is represented by the dotted line. The time needed to kill 50% of infected larvae (LT_50_) was significantly different between the two strains (*p* < 0.001, Wilcoxon test).

The insect virulence of the *P. luminescens* strain overexpressing *dam* was compared to that of the control strain. It was assessed by injection of bacterial cells in *S. frugiperda* (Figure 5). Both strains were pathogenic, being able to cause death of all injected larvae in <60 h. However, while the time needed to kill 50% of infected larvae (LT_50_) was about 38 h for the control strain, it was significantly increased in *P. luminescens* pBB-dam, reaching 44.5 h (*p* < 0.001, Wilcoxon test). This delay in killing insect larvae indicates a reduction in virulence properties of the *P. luminescens* strain overexpressing *dam*.

After loss of the *dam* overexpressing plasmid as described above, cured strains displayed a significantly lower LT_50_ compared to that of the *dam* overexpressing *P. luminescens* strain (data not shown), confirming that *dam*-overexpression causes an impaired virulence in *P. luminescens*.

### Flagellar genes are downregulated in the *P. luminescens dam*-overexpressing strain

**Figure 6:**
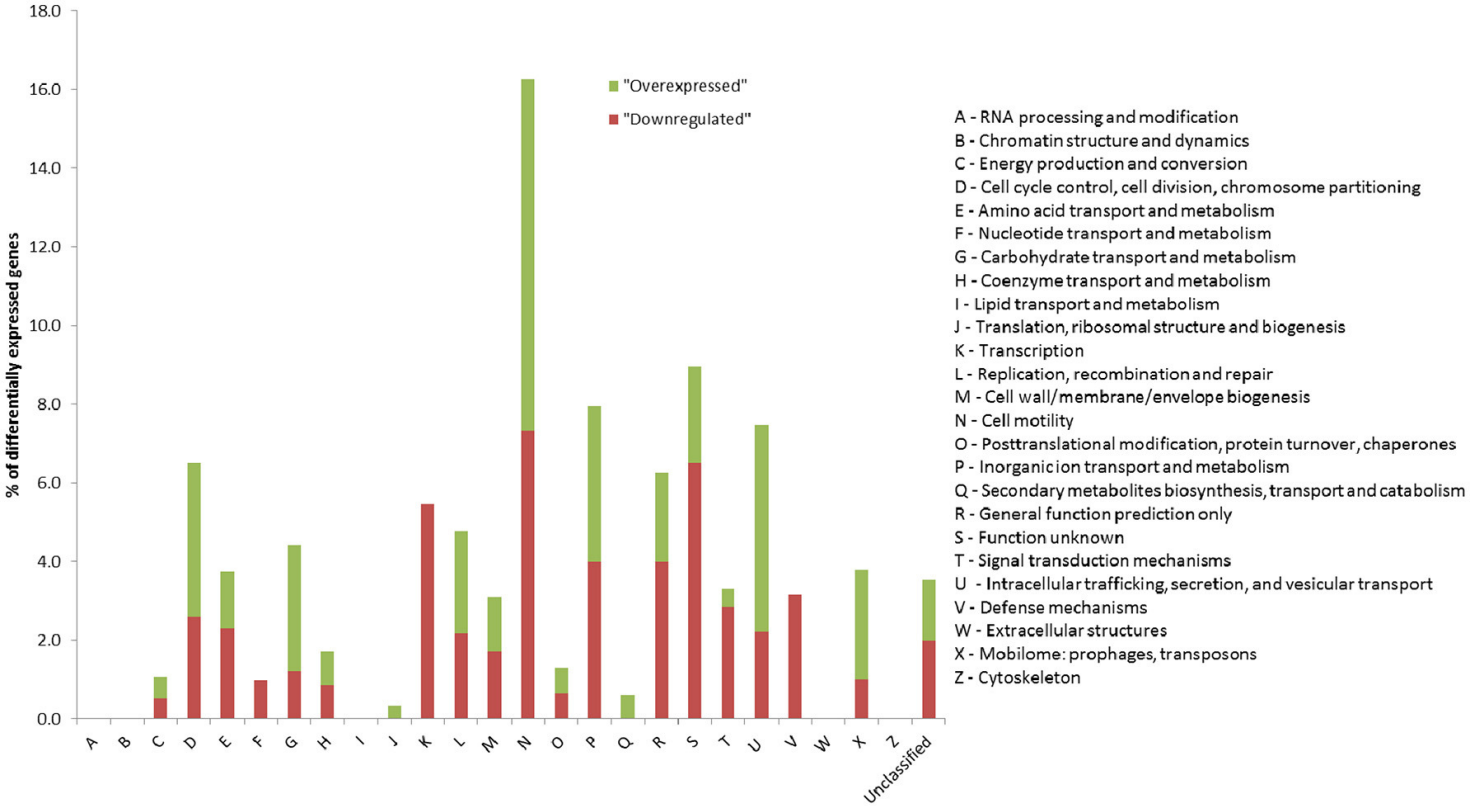
Classification by COG (cluster of orthologous group) annotation of the 208 genes differentially expressed between *P. luminescens* overexpressing *dam* and the control strain. Results show the percentage of genes from each COG class differentially expressed between the *P. luminescens dam*-overexpressing strain and the control strain, according to the 2014 update (ftp://ftp.ncbi.nih.gov/pub/COG/COG2014/static/lists/homeCOGs.html).

We wondered if the reduced motility and the delayed virulence in insects observed for the *P. luminescens* strain overexpressing *dam* were associated to changes in gene expression. RNA sequencing was therefore performed on the *dam* overexpressing strain and the control strain during exponential phase of growth. The transcriptome analysis revealed significant differences (log2 fold change ≥1; adjusted *p* ≤ 0.005) in expression for 208 genes between the two strains, with 121 down-regulated and 87 up-regulated genes in the *P. luminescens* strain overexpressing *dam* (Table S2). Both up- and down-regulated genes were distributed all over the chromosome (Table S2). The percentages by COG class in TT01 genome revealed that the 208 differentially expressed genes belong to various COG categories (Figure 6). Interestingly, we observed an enrichment in genes encoding proteins belonging to the N category, and putatively involved in “cell motility” (Figure 6 and Table S2). Most of them are either flagellar genes or encode putative pili/fimbrial proteins (Table S2). When focusing on the 49 flagellar genes that are found in the *P. luminescens* TT01 genome, 48 of them displayed a lower expression in the strain overexpressing *dam* when compared to the control strain, although not always reaching a significant adjusted *p*-value to be considered as differentially expressed (Table S3). Interestingly, 16 genes encoding putative pili or fimbrial proteins were upregulated in the *dam*-overexpressing strain (Table S2), including *pilL* (plu1049), the first gene of an operon encoding a type IV pilus. This result may explain the observed increased biofilm formation in this strain.

However, while an impaired virulence was observed in the *P. luminescens* strain overexpressing *dam*, only a limited number of genes (*n* = 5) encoding effectors influencing the infection process were differentially regulated (Table S2). In order to confirm these observation, a few genes related to the two major impaired phenotypes (i.e., motility and insect virulence) were therefore selected and their level of expression was quantified by RT-qPCR in the *P. luminescens* strain overexpressing *dam* in comparison to the control strain. As mentioned above, the *dam* gene was up-regulated about 23-fold. Results also showed that 7 of the 10 tested flagellar genes were significantly downregulated in the *dam*-overexpressing strain (Figure S2), in agreement with the results of the RNA-seq analysis. This result confirmed that a reduced expression at the transcriptional level of flagellar genes was responsible for the impaired motility. In contrast, 5 additional genes (*manA, sodA, luxS, tcaZ, lopT*) likely involved in insect virulence displayed similar level of expression between the two strains (Figure S2). Finally, RT-qPCR confirmed the overexpression of a fimbrial gene (*madA*) in the *dam*-overexpressing strain (Figure S2).

## Discussion

Given the high degree of conservation of Dam methyltransferase among enterobacteria and several other Gram-negative bacteria (Lobner-Olesen et al., 2005; Casadesus and Low, 2013; Marinus and Løbner-Olesen, 2014), the identification of a *dam* ortholog in *P. luminescens* was expected. We first showed that the amino-acids previously described as essential for the Dam function in several organisms (Erova et al., 2006a; Horton et al., 2006) were conserved in the predicted amino-acid sequence of the plu0087 *dam* orthologous gene. This result strongly suggested that the predicted encoded enzyme, M.PluTDamP, has the same DNA methylation function in *P. luminescens* as that described for Dam proteins from other Gram-negative bacteria. The finding that the *P. luminescens dam* gene was able to complement the *E. coli dam* mutant, confirmed that M.PluTDamP was able to induce DNA-adenine methylation of GATC sites. Since our results confirmed the putative function of M.PluTDamP, the enzyme should now be named M.PluTDam (or M.PluTII) (Roberts et al., 2003).

The pleiotropic role of DNA-methylation by Dam has been illustrated in many bacterial species (Marinus and Casadesus, 2009). In some of them, it was proposed an essential function of *dam* for cell viability (Julio et al., 2001; Erova et al., 2006b). In particular, the role of Dam during bacterial-host interactions has been reported in several bacterial species (Heusipp et al., 2007), and mostly in the mammalian pathogen *Salmonella* (Garcia-Del Portillo et al., 1999; Heithoff et al., 1999), but also in *E. coli, H. influenzae, Mycobacterium tuberculosis, Campylobacter, Klebsiella, Actinobacillus, Yersinia pseudotuberculosis*, and *Y. pestis* (Julio et al., 2001; Watson et al., 2004; Robinson et al., 2005; Wu et al., 2006; Mehling et al., 2007; Kim et al., 2008; Shell et al., 2013). However, the role of DNA methylation in host-pathogen interaction remains unexplored in bacterial insect pathogens. Construction of a *P. luminescens dam*-mutant failed despite several attempts, suggesting that the presence of Dam itself is possibly required for some essential mechanisms, as described in other bacterial species (Julio et al., 2001; Erova et al., 2006b; Demarre and Chattoraj, 2010). We therefore investigated the role of Dam in *P. luminescens* by using a strain overexpressing *dam*. Growth in standard conditions together with the observation of most major phenotypes were found unmodified by the 23-fold increase of *dam* expression. Remarkably, two major phenotypes were impaired when compared to the control strain: motility and virulence properties were significantly reduced in *P. luminescens* overexpressing *dam. P. luminescens* harbors a large repertoire of factors involved in bacterial-host interaction (Clarke, 2008), and several genes have been described to contribute to insect virulence (Nielsen-LeRoux et al., 2012). For instance, isogenic mutants of genes encoding the SodA or the LuxS proteins display reduced virulence (Krin et al., 2006; Chalabaev et al., 2007). The Tc toxins and the type III secretion system (T3SS) are also considered to play key roles during *P. luminescens*-insect interactions (Bowen et al., 1998; Brugirard-Ricaud et al., 2005; Gatsogiannis et al., 2013). In addition, a *manA*-mutant is impaired in motility, insect virulence, but also in biofilm formation (Amos et al., 2011). Despite a significantly impaired virulence in *P. luminescens* overexpressing *dam*, no change in gene expression was detected for *manA, sodA, luxS, tcaZ, lopT* (encoding an effector of the T3SS), neither by RNA-seq nor RT-qPCR analysis. Thus, the precise factors involved in the observed impaired virulence of the *P. luminescens* strain overexpressing *dam* remains to be elucidated.

Our findings suggest that genome-wide alterations of methylation states may significantly impact some major phenotypes, although the specific mechanisms by which DNA methylation regulates the expression of the genes involved in these phenotypes remains unknown in *P. luminescens*. Strikingly, there is no GATC site in the promoter region of *flhD*, the gene encoding the master regulator of flagellar cascade. Therefore, the *flhD* downregulation (and consequently, of other flagellar genes) in the Dam overexpressing strain cannot be directly related to a difference in GATC methylation states in this locus. However, the observed downregulation of flagellar genes in *P. luminescens dam*-overexpressing strain opens new fields of investigation. The impaired motility coupled with an impaired ability to kill insects identified in this study may illustrate the occurrence of a direct mechanism, yet undescribed, involving these two phenotypes. Indeed, a complex interplay between the regulation of flagellar motility and the expression of virulence factors exists in several bacterial pathogens (Josenhans and Suerbaum, 2002). In particular, these two phenotypes were previously shown as being linked in the closely related genus *Xenorhabdus* (Givaudan and Lanois, 2000, 2017). In this bacterium, the global regulators encoded by the class I operon *flhDC*, controls the expression of class II genes, including most of the structural genes for the flagellar hook-basal body, but also the *fliAZ* operon encoding the alternative sigma factor FliA and another flagellar regulator, FliZ (Givaudan and Lanois, 2000, 2017; Park and Forst, 2006). FliZ was found to upregulate many genes, including genes encoding 2 hemolysins, and a toxin complex (Tc) protein, all of them being considered as virulence factors (Lanois et al., 2008). In *P. luminescens*, no mutant in flagellar regulators has been described (Givaudan and Lanois, 2017). However, the mutation of a response regulator of a two-component system (i.e., AstR) in *P. luminescens* TT01, which regulates the *flhDC* transcription level, causes an impaired motility but has no impact on virulence in insects (Derzelle et al., 2004a). In addition, it was shown that two distinct flagellar genes (*flgG* and *motAB*) deletion mutants, that were consequently non-motile, were as efficient as their parental strain in killing insects (Easom and Clarke, 2008). Our result revealed that *dam* overexpression causes both a reduced motility linked to a downregulation of flagellar genes (i.e., *flhDC, motAB…*) and an impaired virulence. Thus, further investigations are required in *P. luminescens* in order to determine if a common regulator (such as FlhD, FlhC, FliA, or FliZ) is involved in both motility and the ability to kill insects, as already described in *X. nematophila*. Then, it will be important to determine if the changes in the global methylation state caused by *dam*-overexpression involve such common regulator, or in contrast if it impairs distinct mechanisms that are involved in each of these two phenotypes.

A differential expression of several genes encoding virulence factors such as *pap, agn43, sci1* in *E.coli* (Blyn et al., 1990; Henderson and Owen, 1999; Brunet et al., 2011), but also *gtr*, or *opvAB* in *Salmonella* (Broadbent et al., 2010; Cota et al., 2016), is related to a differential methylation state of the GATC sites found in their respective promoters. This is caused mostly by differences in DNA affinity, depending on the DNA methylation state, of various transcriptional repressors (OxyR, Lrp, or Fur). In *X. nematophila*, virulence attenuation in insects was shown to be associated with an *lrp* mutation and Lrp positively regulates the expression of the *flhD* gene encoding the master flagellar regulator (Cowles et al., 2007; Lanois et al., 2008). Thus, because of the presence of *lrp* (plu1600) but also of *oxyR* (plu4740) and *fur* (plu1327) orthologs in the *P. luminescens* genome (Duchaud et al., 2003), it remains to be determined if similar mechanisms exist in *P. luminescens*.

It was shown that GATC methylation itself but also the level of Dam, have multiple functions in the cell (Low and Casadesus, 2008). These functions are correlated with three DNA transactions: DNA mismatch repair, initiation of chromosome replication, and regulation of gene expression (Marinus and Løbner-Olesen, 2014). Our results confirm that modifying the *dam* level of expression in *P. luminescens* causes major phenotypes, presumably in the regulation of gene expression. However, *dam* overexpression did not significantly cause difference in the growth rate (Figure 2). In addition, this overexpression did not lead to the identification of drug-induced mutators in the tested conditions (Table 2). This is in contrast to what was described in other species (Chen et al., 2003) for which the general mutation rate is however similar to what is observed in *P. luminescens*. Our result suggests that after DNA replication, the Mismatch Repair (MMR) apparatus is highly efficient and therefore still able to discriminate between the nascent (error-containing) DNA strand and the mother strand. Many genes putatively involved in regulation of the chromosome replication, in MMR (including *mutH*, the gene encoding the nuclease which relies on the DNA methylation state to identify the correct strand), in double strand breaks (DSBs) repair and/or required for viability of *dam* mutants in other bacterial species are conserved in *P. luminescens* TT01 (Table S4). Such mechanisms may require the presence of Dam for cell viability in *P. luminescens* during the growth conditions tested. In contrast, our results suggest that an elevated level of *dam* expression does not drastically affect the mechanisms involved in chromosomal replication or in DNA mismatch repair.

This study revealed that DNA methylation state on GATC sites seems to be critical in *P. luminescens* for phenotypes involving interactions with the insect host. Eleven additional MTases are found in the genome and probably contribute to the global DNA methylation state which may also account for some of the *P. luminescens* phenotypes during bacterial-insect interaction.

## Ethics statement

According to the EU directive 2010/63, this study is exempt from the above requirements because experiments were performed on invertebrates animals (insects).

## Author contributions

AP, AL, AG, and JB analyzed the data and designed the experiments; AP performed the experiments; MG and ED performed the RNA-seq; DR and SC analyzed the RNA-seq data; JB drafted the manuscript. All authors revised the manuscript and have approved its final version.

### Conflict of interest statement

The authors declare that the research was conducted in the absence of any commercial or financial relationships that could be construed as a potential conflict of interest.
