# Supplementary material for: DNA Adenine Methyltransferase (Dam) Overexpression Impairs Photorhabdus luminescens Motility and Virulence
Source: Front Microbiol. 2017 Sep 1;8:1671. doi: 10.3389/fmicb.2017.01671 (PMC5585154; doi:10.3389/fmicb.2017.01671)
Supplement: Supplementary file 1 [file Table1.PDF]

Table S1: Primers used in this study.

Oligonucleotides	use	Sequence (5’- 3’)[a]
Cp-plu087-F	Cloning of *dam* gene	GGCGGTACCCATGAAAAAAAAACGCGCTTTC
Cp-plu087-R	Cloning of *dam* gene	TCCCGGATCCTCATCCCTTAATTGCCAAGC
plu0004-F	qRT-PCR on *gyrB* gene	ATACACGAAGAAGAAGGTGTTTCAG
plu0004-R	qRT-PCR on *gyrB* gene	TACCTGTCTGTTCAGTTTCTCCAAC
plu4089-F	qRT-PCR on *fis* gene	TAAATTCTGACGTACTAACCGTTGC
plu4089-R	qRT-PCR on *fis* gene	CCAATACCAGCTCATACAGATCATT
plu087-F	qRT-PCR on *dam* gene	CTGATGAGTTTATAGTCCACACACG
plu087-R	qRT-PCR on *dam* gene	ACCATGAGAATTATAACGACAGAGG
plu1847-F	qRT-PCR on *flhD* gene	TGAGTACGGTTGAATTGCTCA
plu1847-R	qRT-PCR on *flhD* gene	TCACTCTCTTCGAACCGGAA
plu1848-F	qRT-PCR on *flhC* gene	ACAATTGGCGATGGAACTCA
plu1848-R	qRT-PCR on *flhC* gene	CTGTTCCCACGTCATAAACCA
plu1850-F	qRT-PCR on *motB* gene	GCGTAACGTCTCAGTCATTAGG
plu1850-R	qRT-PCR on *motB* gene	TCTTGTGGGCTGGAAATTGC
plu1857-F	qRT-PCR on *cheY* gene	TGGCGAATAAGGATCTGAGATT
plu1857-R	qRT-PCR on *cheY* gene	CCCCATCCTGAGCTTCTTCT
plu1912-F	qRT-PCR on *flgN* gene	ACTCGAACAGCAAGTGACCT
plu1912-R	qRT-PCR on *flgN* gene	GCGCGGACAACAGGAAAATT
plu1916-F	qRT-PCR on *flgC* gene	GCTCGGCATTATCAGCTCAA
plu1916-R	qRT-PCR on *flgC* gene	CAACACGGAAAACCACCTGT
plu1936-F	qRT-PCR on *fliR* gene	CTGATCCAGTTCGTCAGTGAA
plu1936-R	qRT-PCR on *fliR* gene	GGTTGCAGGTTTGGCATCAA
plu1942-F	qRT-PCR on *fliL* gene	ACTGTTAGTACTGATTGCCGT
plu1942-R	qRT-PCR on *fliL* gene	ATGGCTTTAGATGCCGCAGA
plu1954-F	qRT-PCR on *fliC* gene	CTTGAATAGATCCCAGGGTACTTTG
plu1954-R	qRT-PCR on *fliC* gene	TAGAGATACCGTCATTAGCGTTACG
plu1956-F	qRT-PCR on *fliZ* gene	CAACACAAAAGAAACGGCCG
plu1956-R	qRT-PCR on *fliZ* gene	TCAATCAACTCCGTCATCTCG
plu0261-F	qRT-PCR on *madA* gene	AATGCGGCGAATAATGCCAT

plu0261-R	qRT-PCR on *madA* gene	TTTGAAATTCCTGCGCCTGG
plu0075-F	qRT-PCR on *sodA* gene	CCTCATTTTGATAAGCAGACGATGG
plu0075-R	qRT-PCR on *sodA* gene	AGTTGTGCCTAATTTCAGACCTTTC
plu0514-F	qRT-PCR on *tcaZ* gene	ATGGCATTAAAAGGTGTGATTGCTG
plu0514-R	qRT-PCR on *tcaZ* gene	CAAATCGAGGAATTTTAGTCAGGGG
plu1253-F	qRT-PCR on *luxS* gene	CTGTTGACCATACTCGAATGAATGC
plu1253-R	qRT-PCR on *luxS* gene	AATAATTTCAACGCCCTCACCATTG
Plu2360-F	qRT-PCR on *manA* gene	AGTTTCAGATAGCTTTCACCGTTTG
Plu2360-R	qRT-PCR on *manA* gene	AAGGGGTAAGGGCATAAACCAATTC
plu3788-F	qRT-PCR on *lopT* gene	GTAAATCCCTGAAAGTCTCTTCCCC
plu3788-R	qRT-PCR on *lopT* gene	AGAATTTCGAACACTCCCTAACGTG
upF-plu0087	Disruption of *dam* gene	TGGACTGCAGCGGCTAAATCAAAACCATCG
upR-plu0087	Disruption of *dam* gene	TGGTGGATCCCGCCAACAAATGGCTCTATC
dnF-plu0087	Disruption of *dam* gene	TGGTGGATCCCGGGAGCAATTTAACCAAAG
dnR-plu0087	Disruption of *dam* gene	TGGATCTAGATCATCCCTTAATTGCCAAGC

[a] Restriction enzyme sites are underlined
