# Supplementary material for: DNA Adenine Methyltransferase (Dam) Overexpression Impairs Photorhabdus luminescens Motility and Virulence
Source: Front Microbiol. 2017 Sep 1;8:1671. doi: 10.3389/fmicb.2017.01671 (PMC5585154; doi:10.3389/fmicb.2017.01671)
Supplement: Supplementary file 2 [file Table2.PDF]

Supplementary Table S2 : List of 208 genes differentially expressed between the Dam-overexpressing P. luminescens TT01 strain and its control strain (LB culture, logarithmic phase, FDR≤0.005, abs(log2FC)≥1)

* ratio Dam+/control (blue, overexpressed in Dam+ strain; red, underexpressed in Dam+ strain)

Highlighted functions:

flagellar genes (13 genes differentially regulated / 49 genes in the genome)

pili/fimbriae genes (16 genes differentially regulated /76 genes in genome), as defined by Duchaud et al 2003

** Mutation of these genes in *Photorhabdus* (or their ortholog in *Xenorhabdus)* influences the infection process in lepidopteran larvae (as described by Nielsen-Leroux *et al* ., 2012)

"N" functionnal category (COG classification)

the *dam* gene is in bold type

											Primary COG_ID				Secondary COG_ID (if any)			
Label	Type	Name	Product	Begin	End	Length	Frame	normalized average read count	log2 fold change *	adjusted pvalue (FDR)	COG #	Functionnal categories (letter)	Functionnal categories (detailed)	Broad functionnal category	COG #	Functionnal categories (letter)	Functionnal categories (detailed)	Broad functionnal category
plu0012	CDS	pts23	Phage tail protein	14517	14636	120	-2	205	1.5	1.51E-03	Unknow							
plu0013	CDS	pts22	Phage tail protein	14651	14968	318	-3	493	1.68	7.65E-05	Unknow							
plu0014	CDS	pts21	Major phage tail tube protein	14995	15510	516	-1	771	1.7	3.11E-05	COG3498	X	Mobilome: prophages, transposons					
plu0015	CDS	pts20	Major phage tail sheath protein	15521	16693	1173	-3	1173	1.87	1.92E-06	COG3497	X	Mobilome: prophages, transposons					
plu0022	CDS	pts13	Phage tail protein	20646	21260	615	-2	260	1.04	3.79E-02	COG4385	X	Mobilome: prophages, transposons					
plu0024	CDS	pts11	Phage baseplate assembly protein	22256	22597	342	-3	149	1.15	3.78E-02	COG3628	X	Mobilome: prophages, transposons					
plu0025	CDS	pts10	Phage-related baseplate assembly protein	22597	23439	843	-1	128	1.42	7.08E-03	COG4540	X	Mobilome: prophages, transposons					
plu0052	CDS	asnA	Aspartate--ammonia ligase (Asparagine synthetase A)	46030	47022	993	1	10400	-1.4	7.16E-03	COG2502	E	Amino acid transport and metabolism	METABOLISM				
**plu0087**	**CDS**	**dam**	**DNA adenine methylase (Deoxyadenosyl-methyltransferase)**	81849	82661	813	-2	17800	5.27	1.64E-24	COG0338	L	Replication, recombination and repair	INFORMATION STORAGE AND PROCESSING				
plu0136	CDS	-	conserved hypothetical protein	143880	144713	834	3	180	1.22	2.21E-02	COG0827	L	Replication, recombination and repair	INFORMATION STORAGE AND PROCESSING				
plu0169	CDS	-	hypothetical protein	180224	180394	171	2	391	-1.12	1.18E-02	Unknow							
plu0180	CDS	-	conserved hypothetical protein	191028	191300	273	-2	554	-1.05	2.93E-02	COG4453	S	Function unknown	POORLY CHARACTERIZED				
plu0224	CDS	-	conserved hypothetical protein	229778	230323	546	-3	227	1.59	1.46E-03	Unknow							
plu0225	CDS	-	Putative TpsA-related protein	230329	235488	5160	-1	3256	1.3	4.52E-03	COG3210	U	Intracellular trafficking, secretion, and vesicular transport					
plu0254	CDS	-	Putative functional antitoxin of the RelE-RelB toxin-antitoxin syst	271540	271794	255	1	389	-1.09	1.75E-02	COG3077	V	Defense mechanisms					
plu0255	CDS	-	putative toxin of the RelE-RelB toxin-antitoxin system; Qin proph	271784	272074	291	2	596	-1.12	9.29E-03	COG2026	V	Defense mechanisms					
plu0261	CDS	madA	fimbrial protein MadA	277866	278471	606	3	387	1.2	1.67E-02	COG3539	N	Cell motility	CELLULAR PROCESSES AND SIGNALING				
plu0263	CDS	madC	fimbrial protein MadC	279376	279948	573	1	255	1.06	3.27E-02	COG3539	N	Cell motility	CELLULAR PROCESSES AND SIGNALING				
plu0264	CDS	madD	fimbrial protein MadD	280016	280738	723	2	342	1.23	8.44E-03	COG3539	N	Cell motility	CELLULAR PROCESSES AND SIGNALING				
plu0279	CDS	-	putative hypothetical secreted protein of Photorhabdus	295804	296493	690	-1	1174	-1.43	2.61E-04	Unknow							
plu0285	CDS	-	conserved hypothetical protein	301670	302125	456	-3	456	-1.56	2.42E-04	COG0328	L	Replication, recombination and repair	INFORMATION STORAGE AND PROCESSING	COG3077	V	Antitoxin component of the RelBE or YafQ-DinJ toxin-antitoxin module	
plu0321	CDS	-	-	345064	345411	348	1	12	1.79	2.46E-02	COG1263	G	Carbohydrate transport and metabolism	METABOLISM				
plu0335	CDS	-	Putative ImpA protein, N-terminal (domain IPR010657)	355899	356792	894	-2	49	1.33	2.77E-02	COG3515	U	Intracellular trafficking, secretion, and vesicular transport					
plu0336	CDS	-	Putative Type VI secretion system IcmF, C-terminal (domain IPR0	356888	360433	3546	-3	120	1.16	3.57E-02	COG3523	U	Intracellular trafficking, secretion, and vesicular transport					
plu0354	CDS	-	Conserved hypothetical protein	377114	377533	420	-3	128	1.19	3.57E-02	COG5435	S	Function unknown	POORLY CHARACTERIZED				
plu0355	CDS	-	Putative Type VI secretion system, RhsGE-associated Vgr protein	377581	379476	1896	-1	258	1.47	8.03E-04	COG3501	UXR						
plu0356	CDS	-	Truncated gene; similarities with putative component of Type VI	379498	380175	678	-1	121	1.3	1.18E-02	COG3515	U	Intracellular trafficking, secretion, and vesicular transport					
plu0417	CDS	-	-	446415	447017	603	-2	302	1.26	1.58E-03	COG3539	N	Cell motility	CELLULAR PROCESSES AND SIGNALING				
plu0418	CDS	-	Major structural subunit MrpA of Proteus mirabilis	447141	447707	567	-2	2094	1.64	2.03E-06	COG3539	N	Cell motility	CELLULAR PROCESSES AND SIGNALING				
plu0470	CDS	malP	maltodextrin phosphorylase	506182	508584	2403	-1	424	1.15	1.84E-02	COG0058	G	Carbohydrate transport and metabolism	METABOLISM				
plu0531	CDS	-	hypothetical protein	586977	587129	153	3	27	1.31	3.03E-02	Unknow							
plu0563	CDS	thrA	aspartokinase I-homoserine dehydrogenase I	636040	638499	2460	1	4389	-1.36	6.35E-03	COG0460	E	Amino acid transport and metabolism	METABOLISM	COG0527	E	Amino acid transport and metabolism	METABOLISM
plu0587	CDS	nhaA	Na(+)/H(+) antiporter 1 (sodium/proton antiporter 1)	668004	669170	1167	3	2037	-1.52	4.32E-04	COG3004	P	Inorganic ion transport and metabolism	METABOLISM				
plu0706	CDS	-	HicB-like protein	816018	816374	357	-2	2086	-1.48	2.36E-04	COG4226	R	General function prediction only	POORLY CHARACTERIZED				
plu0707	CDS	-	HicA-like protein	816371	816646	276	-3	1571	-1.52	3.30E-04	Unknow							
plu0769	CDS	mrfA	Major fimbrial subunit polypeptide, MrfA	888552	889091	540	3	175	1.99	8.19E-06	COG3539	N	Cell motility	CELLULAR PROCESSES AND SIGNALING				
plu0770	CDS	mrfB	Fimbrial pilin protein precursor, MrfE	889175	889723	549	2	101	1.24	3.57E-02	COG3539	N	Cell motility	CELLULAR PROCESSES AND SIGNALING				
plu0786	CDS	-	Conserved hypothetical protein. Probable transmembrane prote	905365	905925	561	1	490	1.98	1.53E-06	COG3539	N	Cell motility	CELLULAR PROCESSES AND SIGNALING				
plu0787	CDS	-	putative fimbrial chaperone	906027	906743	717	3	235	1.17	2.49E-02	COG3121	N	Cell motility	CELLULAR PROCESSES AND SIGNALING	COG3121	U	Intracellular trafficking, secretion, anc	CELLULAR PROCESSES AND SIGNALING
plu0792	CDS	-	conserved hypothetical protein	911804	912958	1155	2	426	1.02	4.48E-02	COG0741	M	Cell wall/membrane/envelope biogenesis	CELLULAR PROCESSES AND SIGNALING				
plu0866	CDS	-	putative proteic killer suppression protein	993308	993589	282	-3	88	-1.11	4.44E-02	COG3549	R	General function prediction only	POORLY CHARACTERIZED				
plu0890	CDS	-	putative pyocin S3 immunity protein	1016619	1017086	468	3	43	-1.48	3.86E-02	Unknow							
plu0922	CDS	-	-	1062305	1063027	723	-3	1141	-1.26	2.03E-03	COG2197	K	Transcription	INFORMATION STORAGE AND PROCESSING	COG2197	T	Signal transduction mechanisms	CELLULAR PROCESSES AND SIGNALING
plu0927	CDS	-	putative MFS transporter	1068465	1069637	1173	-2	110	1.2	3.57E-02	COG2814	G	Carbohydrate transport and metabolism	METABOLISM				
plu0959	CDS	-	conserved hypothetical protein	1098942	1099238	297	-2	107	-1.71	1.30E-04	COG4453	S	Function unknown	POORLY CHARACTERIZED				
plu0997	CDS	phfS	PhfC protein, putative adhesion protein, fimbriae	1181612	1182121	510	-3	490	-1.59	8.83E-05	COG3539	N	Cell motility	CELLULAR PROCESSES AND SIGNALING				
plu1021	CDS	-	-	1203054	1203323	270	-2	32	1.44	3.57E-02	COG3344	X	Mobilome: prophages, transposons					
plu1049	CDS	pilL	-	1227925	1228974	1050	1	174	1.08	3.57E-02	COG3266	D	Cell cycle control, cell division, chromoson	CELLULAR PROCESSES AND SIGNALING				
plu1064	CDS	-	putative DNA-binding prophage protein	1241604	1241915	312	-2	3526	-1.09	3.57E-02	COG3636	K	Transcription	INFORMATION STORAGE AND PROCESSING				
plu1097	CDS	-	Conserved hypothetical protein	1267164	1267526	363	3	61	1.33	4.42E-02	Unknow							
plu1140	CDS	-	conserved hypothetical protein	1320126	1321181	1056	-2	117	2.21	1.77E-06	COG3210	U	Intracellular trafficking, secretion, and vesicular transport					
plu1209	CDS	-	putative membrane protein	1389961	1390977	1017	-1	95	-1.3	1.64E-02	COG4104	U	Intracellular trafficking, secretion, and vesicular transport					
plu1223	CDS	-	conserved hypothetical protein	1416046	1416384	339	-1	11600	-1.16	4.48E-02	COG5606	R	General function prediction only					
plu1224	CDS	-	conserved hypothetical protein	1416365	1416745	381	-3	9118	-1.22	2.93E-02	COG4679	S	Function unknown	POORLY CHARACTERIZED				
plu1342	CDS	-	-	1589606	1590259	654	2	103	1.18	2.32E-02	COG2931	Q	Secondary metabolites biosynthesis, trans	METABOLISM	COG1073	T		
plu1359	CDS	-	-	1621192	1622322	1131	-1	9	2.86	2.80E-04	COG4974	L	Replication, recombination and repair	INFORMATION STORAGE AND PROCESSING				
plu1402	CDS	-	putative transcriptional regulator	1684697	1684981	285	-3	1187	-2.16	1.02E-08	COG2944	K	Transcription	INFORMATION STORAGE AND PROCESSING				
plu1403	CDS	-	conserved hypothetical protein	1684983	1685156	174	-2	1086	-1.86	1.17E-06	COG4737	S	Function unknown	POORLY CHARACTERIZED				
plu1404	CDS	-	conserved hypothetical protein	1685147	1685314	168	-3	628	-1.58	7.68E-05	COG4737	S	Function unknown	POORLY CHARACTERIZED				
plu1476	CDS	modA	molybdate-binding periplasmic protein precursor	1770069	1770839	771	3	604	-1.07	3.03E-02	COG0725	P	Inorganic ion transport and metabolism	METABOLISM				
plu1482	CDS	-	hypothetical protein	1775312	1775986	675	2	298	-1.54	3.47E-04	COG1893	H	Coenzyme transport and metabolism	METABOLISM				
plu1537**	CDS	pit	Insecticidal crystal-related protein Pit	1838326	1838736	411	-1	1041	1.89	3.92E-06	Unknow							
plu1561	CDS	-	Putative calcium-dependent cell adhesion molecule-1 of Dictyost	1866612	1867232	621	-2	114	1.48	7.93E-03	Unknow							
plu1608	CDS	-	conserved hypothetical protein	1921727	1922050	324	-3	73	-1.68	8.03E-04	COG5473	S	Function unknown	POORLY CHARACTERIZED	COG0494	V		
plu1617	CDS	-	conserved hypothetical protein	1933264	1933539	276	1	2587	-1.03	4.70E-02	COG4453	S	Function unknown	POORLY CHARACTERIZED				
plu1641	CDS	-	conserved hypothetical protein	1961833	1962453	621	-1	2159	-1.28	6.90E-03	Unknow							
plu1685	CDS	-	conserved hypothetical protein	2010883	2011341	459	-1	38	1.73	5.69E-03	Unknow							
plu1688	CDS	-	conserved hypothetical protein	2014005	2015087	1083	-2	56	1.49	1.20E-02	COG3497	R	General function prediction only	POORLY CHARACTERIZED				
plu1717	CDS	-	conserved hypothetical protein	2048099	2049754	1656	-3	23	1.43	4.44E-02	COG1196	D	Cell cycle control, cell division, chromoson	CELLULAR PROCESSES AND SIGNALING				
plu1727	CDS	-	conserved hypothetical protein	2061031	2062215	1185	-1	105	1.17	4.77E-02	COG3497	R	General function prediction only	POORLY CHARACTERIZED				
plu1776	CDS	sulA	Cell division inhibitor SulA	2119585	2120118	534	-1	407	-1.04	2.98E-02	COG5404	D	Cell cycle control, cell division, chromoson	CELLULAR PROCESSES AND SIGNALING				
plu1784	CDS	yccV	Hemimethylated oriC DNA-binding protein; weak repressor of dr	2128483	2128803	321	-1	1794	-1.18	1.01E-02	COG3785	O	Posttranslational modification, protein turnover, chaperones					
plu1788	CDS	ISPlu3V	Transposase, IS630 family	2131018	2132043	1026	1	3	-2.5	3.67E-02	COG3335	L	Replication, recombination and repair	INFORMATION STORAGE AND PROCESSING				
plu1808	CDS	-	hypothetical protein	2156934	2157293	360	3	404	-1	4.70E-02	Unknow							
plu1843	CDS	mgtC	-	2190810	2191520	711	3	372	-1.71	6.88E-05	COG1285	S	Function unknown	POORLY CHARACTERIZED				
plu1845	CDS	mgtB	MgtB (fragment)	2192575	2192757	183	1	20	-1.62	3.47E-02	COG0474	P	Inorganic ion transport and metabolism	METABOLISM				
plu1847**	CDS	flhD	flagellar transcriptional activator	2195317	2195670	354	1	131	-1.05	3.80E-02	COG0796	M	Cell wall/membrane/envelope biogenesis	CELLULAR PROCESSES AND SIGNALING				
plu1848	CDS	flhC	flagellum biosynthesis transcription activator	2195674	2196255	582	1	926	-1.15	9.20E-03	Unknow							
plu1849	CDS	motA	chemotaxis protein MotA	2196392	2197279	888	2	253	-1.09	3.57E-02	COG1291	N	Cell motility	CELLULAR PROCESSES AND SIGNALING				
plu1853	CDS	cheD	Methyl-accepting chemotaxis protein I (MCP-I) (Serine chemorec	2201059	2202753	1695	1	508	-1.8	9.68E-06	COG0840	T	Signal transduction mechanisms	CELLULAR PROCESSES AND SIGNALING	COG0840	N	Cell motility	CELLULAR PROCESSES AND SIGNALING

plu1854	CDS	tap	-	2202807	2204441	1635	3	461	-1.11	2.40E-02	COG0840	T	Signal transduction mechanisms	CELLULAR PROCESSES AND SIGNALING				
plu1863	CDS	-	Hypothetical protein	2209346	2209504	159	-3	66	-1.47	6.50E-03	Unknow							
plu1864	CDS	-	Truncated gene. Putative phosphoenolpyruvate phosphomutase	2210011	2210184	174	1	80	-1.22	1.04E-02	COG0615	M	Cell wall/membrane/envelope biogenesis	CELLULAR PROCESSES AND SIGNALING				
plu1865	CDS	-	Truncated gene. Putative phosphoenolpyruvate phosphomutase	2210256	2210996	741	3	59	-1.31	2.54E-02	COG2513	G	Carbohydrate transport and metabolism	METABOLISM				
plu1879	CDS	glbB	Similar to protein encoded in the glidobactin A synthetase gene	2225254	2226519	1266	-1	222	1.14	3.27E-02	COG0477	GEPR						
plu1899	CDS	-	conserved hypothetical protein	2262599	2263042	444	2	20	1.48	3.45E-02	COG1048	C	Energy production and conversion	METABOLISM	COG2075	J		
plu1913	CDS	flgM	Negative regulator of flagellin synthesis (Anti-sigma-28 factor) Fl	2280636	2280938	303	-2	545	-1.11	2.43E-02	COG2747	K	Transcription	INFORMATION STORAGE AND PROCESSING	COG2747	N	Cell motility	CELLULAR PROCESSES AND SIGNALING
plu1924	CDS	flgK	Flagellar hook-associated protein 1 (HAP1)	2289341	2290990	1650	2	400	-1.45	6.74E-04	COG1256	N	Cell motility	CELLULAR PROCESSES AND SIGNALING				
plu1925	CDS	flgL	Flagellar hook-associated protein 3 (HAP3) (Hook-filament juncti	2291065	2292030	966	1	518	-1.63	4.62E-05	COG1344	N	Cell motility	CELLULAR PROCESSES AND SIGNALING				
plu1951	CDS	fliT	Flagellar protein FliT	2317465	2317842	378	-1	66	-1.23	1.84E-02	Unknow							
plu1952	CDS	fliS	Flagellar protein FliS	2317842	2318252	411	-2	105	-1.21	2.13E-02	COG1516	N	Cell motility	CELLULAR PROCESSES AND SIGNALING	COG1516	U	Intracellular trafficking, secretion, anc	CELLULAR PROCESSES AND SIGNALING
plu1953	CDS	fliD	Flagellar hook-associated protein 2 (HAP2) (Filament cap protein	2318265	2319686	1422	-2	630	-2.08	1.76E-08	COG1345	N	Cell motility	CELLULAR PROCESSES AND SIGNALING				
plu1954	CDS	fliC	Flagellin	2319961	2321028	1068	1	5025	-3.35	1.07E-16	COG1344	N	Cell motility	CELLULAR PROCESSES AND SIGNALING				
plu1961	CDS	phaxB1	PhaxB1, similar to binary toxin XaxB of Xenorhabdus nematophil	2331853	2332881	1029	-1	67	-1.64	6.12E-04	COG0419	L	Replication, recombination and repair	INFORMATION STORAGE AND PROCESSING				
plu1962	CDS	phaxA1	PhaxA1, similar to binary toxin XaxA of Xenorhabdus nematophil	2332924	2334162	1239	-1	139	-1.44	8.03E-04	Unknow							
plu1973	CDS	-	hypothetical protein	2347205	2347441	237	2	1041	-1.28	1.39E-03	Unknow							
plu1974	CDS	-	conserved hypothetical protein	2347410	2347739	330	3	2208	-1.16	4.85E-03	COG2026	V	Defense mechanisms					
plu1986	CDS	-	hypothetical protein	2355775	2356209	435	-1	38	-1.31	2.40E-02	COG2771	K	Transcription	INFORMATION STORAGE AND PROCESSING	COG3668	X		
plu2017	CDS	-	-	2385147	2385434	288	3	13	1.65	3.42E-02	COG4104	U	Intracellular trafficking, secretion, and vesicular transport					
plu2031	CDS	-	Conserved hypothetical protein	2401213	2401551	339	1	228	-1.15	2.12E-02	COG1226	P	Inorganic ion transport and metabolism	METABOLISM				
plu2032	CDS	uspG	Universal stress protein G	2401730	2402167	438	2	252	-1.37	1.93E-03	COG0589	T	Signal transduction mechanisms	CELLULAR PROCESSES AND SIGNALING				
plu2077	CDS	-	hypothetical protein	2461846	2462130	285	1	1512	-1.14	1.04E-02	COG4972	NW						
plu2082	CDS	luxB	Alkanal monooxygenase beta chain (luciferase beta subunit)	2466759	2467733	975	3	180	-1.09	3.57E-02	COG2141	C	Energy production and conversion	METABOLISM				
plu2083	CDS	luxE	Acyl-protein synthetase (long-chain-fatty-acid-luciferin-compone	2467795	2468907	1113	1	93	-1.26	2.51E-02	COG1541	H	Coenzyme transport and metabolism	METABOLISM				
plu2143	CDS	-	hypothetical protein	2525733	2526353	621	-2	127	1.21	4.51E-02	Unknow							
plu2171	CDS	ureA	Urease gamma subunit (Urea amidohydrolase)	2557998	2558300	303	3	356	-1.11	3.27E-02	COG0831	E	Amino acid transport and metabolism	METABOLISM				
plu2211	CDS	-	conserved hypothetical protein	2598182	2598637	456	-3	209	-1.55	1.51E-03	COG0328	L	Replication, recombination and repair	INFORMATION STORAGE AND PROCESSING	COG3077	V		
plu2250	CDS	-	conserved hypothetical protein	2645866	2646108	243	1	1342	-1.03	4.32E-02	COG3609	K	Transcription	INFORMATION STORAGE AND PROCESSING				
plu2252	CDS	-	conserved hypothetical protein	2646494	2646784	291	2	322	-1.07	4.42E-02	COG1598	S	Function unknown	POORLY CHARACTERIZED				
plu2265	CDS	-	conserved hypothetical protein	2662679	2662930	252	-3	253	-1.15	1.51E-02	COG2161	D	Cell cycle control, cell division, chromoson	CELLULAR PROCESSES AND SIGNALING				
plu2268	CDS	-	-	2666118	2666579	462	3	106	-1.12	3.50E-02	COG3311	KX						
plu2277	CDS	-	conserved hypothetical protein	2675668	2676039	372	1	770	-1.03	3.56E-02	COG0346	E	Amino acid transport and metabolism	METABOLISM				
plu2307	CDS	-	-	2706888	2707022	135	-2	78	-2.59	2.55E-07	COG1487	R	General function prediction only	POORLY CHARACTERIZED				
plu2308	CDS	-	conserved hypothetical protein	2707235	2707513	279	-3	2211	-1.18	1.56E-02	COG3636	K	Transcription	INFORMATION STORAGE AND PROCESSING				
plu2309	CDS	-	conserved hypothetical protein	2707510	2707812	303	-1	950	-1.66	4.96E-05	COG3657	S	Function unknown	POORLY CHARACTERIZED				
plu2328	CDS	-	-	2747264	2747920	657	-3	229	-1.27	7.54E-03	COG2755	E	Amino acid transport and metabolism	METABOLISM				
plu2329	CDS	-	-	2747927	2748127	201	-3	188	-1.33	7.06E-03	COG4644	X	Mobilome: prophages, transposons					
plu2330	CDS	-	putative the plasmid stabilization ParD protein	2748978	2749232	255	3	309	-1.27	7.08E-03	Unknow							
plu2351	CDS	-	conserved hypothetical protein	2770570	2771403	834	-1	862	-1.01	4.47E-02	COG0702	M	Cell wall/membrane/envelope biogenesis	CELLULAR PROCESSES AND SIGNALING	COG0702	G	Carbohydrate transport and metaboli	METABOLISM
plu2365	CDS	-	putative plasmid stable inheritance protein K	2783921	2784310	390	-3	2178	-1.01	4.18E-02	COG2337	T	Signal transduction mechanisms	CELLULAR PROCESSES AND SIGNALING				
plu2366	CDS	-	putative plasmid stable inheritance protein I	2784255	2784512	258	-2	1913	-1	3.91E-02	COG2336	T	Signal transduction mechanisms	CELLULAR PROCESSES AND SIGNALING				
plu2369	CDS	-	conserved hypothetical protein	2785516	2785719	204	-1	1913	-1.05	3.42E-02	COG5450	K	Transcription	INFORMATION STORAGE AND PROCESSING				
plu2434	CDS	cysB	Cys regulon transcriptional activator	2856686	2857660	975	-3	1222	-1.02	3.02E-02	COG0583	K	Transcription	INFORMATION STORAGE AND PROCESSING				
plu2460**	CDS	tccA2	Insecticidal toxin complex protein TccA2	2895541	2899062	3522	-1	115	1.2	3.57E-02	COG3206	M	Cell wall/membrane/envelope biogenesis	CELLULAR PROCESSES AND SIGNALING	COG1196	D		
plu2461	CDS	-	Similar to exochitinase	2899159	2900793	1635	-1	100	1.24	2.06E-02	COG3325	G	Carbohydrate transport and metabolism	METABOLISM				
plu2480	CDS	ail1	Ail protein precursor of Yersinia	2920701	2921228	528	-2	4614	-1.2	6.50E-03	COG3637	M	Cell wall/membrane/envelope biogenesis	CELLULAR PROCESSES AND SIGNALING				
plu2565	CDS	-	-	3010375	3010692	318	-1	308	-1.08	2.12E-02	COG3042	R	General function prediction only	POORLY CHARACTERIZED				
plu2580	CDS	tyrR	Transcriptional regulatory protein TyrR	3021998	3023581	1584	-3	904	-1.02	3.79E-02	COG3283	K	Transcription	INFORMATION STORAGE AND PROCESSING	COG3283	E	Amino acid transport and metabolism	METABOLISM
plu2638	CDS	pqrA	Regulatory protein PqrA (AraC/XylS family)	3088162	3088527	366	1	407	-1.95	4.29E-07	COG2207	K	Transcription	INFORMATION STORAGE AND PROCESSING				
plu2651	CDS	-	conserved hypothetical protein	3104667	3104993	327	-2	244	-1.39	3.63E-03	COG4453	S	Function unknown	POORLY CHARACTERIZED				
plu2687	CDS	yebZ	-	3190475	3191368	894	-3	1009	-1.12	8.20E-03	COG1276	P	Inorganic ion transport and metabolism	METABOLISM				
plu2688	CDS	yobA	-	3191371	3191757	387	-1	504	-1.27	4.49E-03	COG2372	P	Inorganic ion transport and metabolism	METABOLISM				
plu2689	CDS	ftnA	nonheme ferritin 1	3191952	3192455	504	-2	3697	-1.19	6.82E-03	COG1528	P	Inorganic ion transport and metabolism	METABOLISM				
plu2707	CDS	-	conserved hypothetical protein	3209538	3209798	261	3	14	-1.41	3.79E-02	COG1396	K	Transcription	INFORMATION STORAGE AND PROCESSING	COG1904	G		
plu2819	CDS	-	-	3348696	3349283	588	3	48	-1.23	3.79E-02	COG0127	F	Nucleotide transport and metabolism	METABOLISM				
plu2846	CDS	-	-	3377040	3377321	282	3	1309	-1.1	2.13E-02	COG1396	K	Transcription	INFORMATION STORAGE AND PROCESSING	COG3620	K		
plu2897	CDS	-	hypothetical protein	3425672	3426157	486	-3	102	1.24	1.89E-02	Unknow							
plu2898	CDS	-	conserved hypothetical protein	3426160	3427317	1158	-1	128	1.09	4.47E-02	COG3566	S	Function unknown	POORLY CHARACTERIZED				
plu2899	CDS	-	conserved hypothetical protein	3427321	3428133	813	-1	83	1.33	1.89E-02	COG2369	S	Function unknown	POORLY CHARACTERIZED				
plu2932	CDS	-	-	3446861	3447283	423	2	11	2.33	2.46E-03	Unknow							
plu2933	CDS	-	Similar to bacteriophage protein	3447353	3448255	903	2	39	1.33	3.57E-02	Unknow							
plu2946	CDS	-	hypothetical protein	3454621	3454839	219	1	5	2.1	1.89E-02	Unknow							
plu2998	CDS	cbiB	CbiB protein	3498179	3499138	960	-3	210	1.24	1.18E-02	COG1270	H	Coenzyme transport and metabolism	METABOLISM				
plu3003	CDS	-	conserved hypothetical protein	3504922	3505212	291	1	1320	-1.08	2.92E-02	COG3093	R	General function prediction only	POORLY CHARACTERIZED				
plu3014	CDS	-	putative phage protein	3514650	3515003	354	3	19	-1.58	1.84E-02	COG1598	S	Function unknown	POORLY CHARACTERIZED				
plu3021	CDS	-	putative phage protein	3522745	3523173	429	-1	88	-1.14	4.70E-02	COG1598	S	Function unknown	POORLY CHARACTERIZED				
plu3057	CDS	-	putative plasmid stability protein (pilT domain)	3561555	3561974	420	-2	2454	-1.05	4.17E-02	COG1487	R	General function prediction only	POORLY CHARACTERIZED	COG0615	M		
plu3058	CDS	-	putative plasmid stabilization protein	3561971	3562210	240	-3	1152	-1.03	3.89E-02	COG4691	R	General function prediction only	POORLY CHARACTERIZED				
plu3101	CDS	-	conserved hypothetical protein	3619962	3620591	630	-2	60	1.51	1.15E-02	Unknow							
plu3109	CDS	astA	arginine N-succinyltransferase (AOST)	3630459	3631490	1032	-2	166	1.65	1.45E-04	COG3138	E	Amino acid transport and metabolism	METABOLISM				
plu3110	CDS	argM	succinylornithine transaminase (succinylornithine aminotransfer	3631512	3632720	1209	-2	185	1.05	3.47E-02	COG4992	E	Amino acid transport and metabolism	METABOLISM				
plu3156	CDS	-	-	3715712	3716482	771	-3	83	-1.46	1.02E-02	Unknow							
plu3176**	CDS	tccZ2	-	3736581	3736823	243	-2	98	-1.94	3.42E-05	Unknow							
plu3253	CDS	-	Putative Type VI secretion system, RhsGE-associated Vgr protein	3852594	3855122	2529	-2	211	1.17	1.79E-02	COG3501	UXR						
plu3255	CDS	-	Putative Outer membrane protein, (OmpA/MotB domain IPR006	3855809	3857536	1728	-3	531	1.19	7.72E-03	COG2885	M	Cell wall/membrane/envelope biogenesis	CELLULAR PROCESSES AND SIGNALING				
plu3260	CDS	-	putative Type VI secretion protein, VC_A0114 (IPR010263)	3860551	3861900	1350	-1	1310	-1.14	2.54E-02	COG3522	U	Intracellular trafficking, secretion, and vesicular transport					
plu3261	CDS	-	putative type VI secretion protein, EvpB/VC_A0108, tail sheath ((	3861916	3863442	1527	-1	2248	-1.92	3.92E-06	COG3517	U	Intracellular trafficking, secretion, and vesicular transport					
plu3262	CDS	-	putative Type VI secretion system, VipA, VC_A0107 or Hcp2 (don	3863474	3863971	498	-3	806	-2.33	2.97E-09	COG3516	U	Intracellular trafficking, secretion, and vesicular transport					
plu3331	CDS	-	Weakly similar to putative P2 tail fiber protein H and RNA polym	3961157	3961984	828	2	69	1.36	3.35E-02	COG4675	S	Function unknown	POORLY CHARACTERIZED	COG5301	X		
plu3333	CDS	-	conserved hypothetical protein	3962809	3963528	720	1	21	1.63	1.90E-02	COG4675	S	Function unknown	POORLY CHARACTERIZED	COG5301	X		
plu3397	CDS	-	hypothetical protein	4020812	4021183	372	-3	91	1.15	4.70E-02	Unknow							
plu3401	CDS	-	conserved hypothetical protein	4022419	4023453	1035	-1	209	1.09	3.45E-02	COG4834	S	Function unknown	POORLY CHARACTERIZED				
plu3402	CDS	-	conserved hypothetical protein	4023453	4023929	477	-2	105	1.29	2.55E-02	COG3391	R	General function prediction only	POORLY CHARACTERIZED				
plu3469	CDS	-	conserved hypothetical protein	4071256	4072455	1200	-1	1326	-1.17	7.93E-03	COG1396	K	Transcription	INFORMATION STORAGE AND PROCESSING	COG2856	E	Amino acid transport and metabolism	METABOLISM
plu3470	CDS	-	hypothetical protein	4072471	4072938	468	-1	867	-1.62	4.62E-05	Unknow							
plu3491	CDS	-	conserved hypothetical protein	4086678	4087145	468	-2	7649	-1.75	1.08E-04	COG1396	K	Transcription	INFORMATION STORAGE AND PROCESSING	COG1598	S	Function unknown	POORLY CHARACTERIZED
plu3507	CDS	-	Putative major fimbrial sub-unit protein	4099071	4099475	405	-2	17	-2.64	4.62E-05	COG3539	N	Cell motility	CELLULAR PROCESSES AND SIGNALING				
plu3559	CDS	-	-	4193422	4193574	153	-1	17	1.46	4.70E-02	COG0477	GEPR						
plu3593	CDS	-	conserved hypothetical protein	4235193	4236086	894	-2	36	1.4	4.34E-02	COG3210	U	Intracellular trafficking, secretion, and vesicular transport					
plu3629	CDS	-	hypothetical protein	4279634	4279777	144	2	6	1.86	1.36E-02	Unknow							
plu3633	CDS	-	-	4281700	4281996	297	1	82	1.34	1.75E-02	COG3539	N	Cell motility	CELLULAR PROCESSES AND SIGNALING				
plu3634	CDS	-	putative the N-terminal region of outer membrane usher protein	4282079	4283065	987	2	159	1.18	2.49E-02	COG3188	N	Cell motility	CELLULAR PROCESSES AND SIGNALING	COG3188	U	Intracellular trafficking, secretion, anc	CELLULAR PROCESSES AND SIGNALING
plu3672	CDS	leuO	activator protein in leuABCD operon	4323714	4324658	945	-2	397	-1.12	1.87E-02	COG0583	K	Transcription	INFORMATION STORAGE AND PROCESSING				
plu3755**	CDS	lopD	Translocator protein LopD	4425401	4426285	885	-3	1152	-1.01	4.51E-02	Unknow							

plu3756	CDS	lopB	Translocator proteins LopB	4426297	4427475	1179	-1	2052	-1.01	4.77E-02	COG5613	S	Function unknown	POORLY CHARACTERIZED				
plu3856	CDS	amtB	Probable ammonium transport protein AmtB	4529787	4531106	1320	-2	220	1.06	3.45E-02	COG0004	P	Inorganic ion transport and metabolism	METABOLISM				
plu3938	CDS	-	conserved hypothetical protein	4623441	4624040	600	3	573	-1.21	7.51E-03	Unknow							
plu4172	CDS	-	putative pyocin S3 immunity protein and to Photorhabdus lumin	4878614	4879081	468	-3	532	-1.24	4.49E-03	Unknow							
plu4173	CDS	-	-	4879191	4879652	462	-2	196	-1.05	3.57E-02	Unknow							
plu4217	CDS	-	Conserved hypothetical protein. Putative membrane protein	4932024	4932692	669	-2	104	-1.17	4.48E-02	Unknow							
plu4233	CDS	-	putative membrane protein	4956862	4957422	561	-1	331	-1.04	3.67E-02	COG0705	R	General function prediction only	POORLY CHARACTERIZED				
plu4248	CDS	osmY	Osmotically inducible protein Y precursor	4970837	4971214	378	-3	350	-1.28	4.83E-03	COG2823	R	General function prediction only	POORLY CHARACTERIZED				
plu4267	CDS	-	-	4992386	4993351	966	2	7	1.77	2.49E-02	COG0683	E	Amino acid transport and metabolism	METABOLISM				
plu4352	CDS	yeiC	Putative kinase YeiC protein of Escherichia coli	5085038	5086123	1086	-3	36	1.39	4.42E-02	COG0524	G	Carbohydrate transport and metabolism	METABOLISM				
plu4446	CDS	fecA	Iron(III) dicitrate outer membrane transporter precursor protein	5194611	5196971	2361	3	3299	1	3.89E-02	COG4772	P	Inorganic ion transport and metabolism	METABOLISM	COG1629	P	Inorganic ion transport and metabolis	METABOLISM
plu4448	CDS	fecC	Iron(III) dicitrate transport system permease protein FecC	5197936	5198934	999	1	352	1.19	1.55E-02	COG0609	P	Inorganic ion transport and metabolism	METABOLISM				
plu4449	CDS	fecD	Iron(III) dicitrate transport system permease protein FecD	5198931	5199887	957	3	306	1.31	4.34E-03	COG0609	P	Inorganic ion transport and metabolism	METABOLISM				
plu4468	CDS	-	hypothetical protein	5221667	5221978	312	-3	43	1.22	4.48E-02	Unknow							
plu4470	CDS	-	putative integrase	5222567	5223433	867	2	102	1.54	4.06E-03	COG0582	L	Replication, recombination and repair	INFORMATION STORAGE AND PROCESSING	COG1516	O	Posttranslational modification, protei	CELLULAR PROCESSES AND SIGNALING
plu4471	CDS	-	-	5223587	5224729	1143	2	350	1.09	2.95E-02	COG3598	L	Replication, recombination and repair	INFORMATION STORAGE AND PROCESSING				
plu4612	CDS	-	-	5376454	5376708	255	-1	109	1.33	1.89E-02	COG3385	X	Mobilome: prophages, transposons					
plu4694	CDS	smf	Smf protein	5478193	5479278	1086	-1	1297	1.04	3.29E-02	COG0758	L	Replication, recombination and repair	INFORMATION STORAGE AND PROCESSING	COG0758	U	Intracellular trafficking, secretion, anc	CELLULAR PROCESSES AND SIGNALING
plu4731	CDS	coaA	pantothenate kinase (pantothenic acid kinase)	5502515	5503465	951	2	235	1.19	2.86E-02	COG1072	H	Coenzyme transport and metabolism	METABOLISM				
plu4748	CDS	-	Conserved hypothetical protein; putative secreted protein	5528419	5529000	582	1	1225	1.46	6.20E-04	Unknow							
plu4781	CDS	-	conserved hypothetical protein	5561171	5561497	327	2	1407	-1.3	1.62E-03	COG4679	S	Function unknown	POORLY CHARACTERIZED				
plu4782	CDS	-	conserved hypothetical protein	5561494	5561832	339	1	420	-1.28	9.55E-04	COG5606	R	General function prediction only	POORLY CHARACTERIZED				
plu4786	CDS	-	-	5563680	5563865	186	3	334	-2.22	6.41E-08	COG3549	R	General function prediction only	POORLY CHARACTERIZED				
plu4787	CDS	-	-	5563807	5563959	153	1	424	-2.25	1.17E-08	COG3549	R	General function prediction only	POORLY CHARACTERIZED				
plu4788	CDS	-	putative proteic killer active protein Hig B of plasmid Rts1	5563959	5564273	315	3	2006	-1.98	4.53E-07	COG3093	R	General function prediction only	POORLY CHARACTERIZED				
plu4789	CDS	-	-	5564298	5564486	189	3	470	-2.45	7.75E-10	COG2944	K	Transcription	INFORMATION STORAGE AND PROCESSING				
plu4790	CDS	-	putative bacteriophage integrase	5564508	5564930	423	3	534	-2.36	2.97E-09	COG0582	L	Replication, recombination and repair	INFORMATION STORAGE AND PROCESSING				
plu4796	CDS	wblA	UDP-glucose/GDP-mannose dehydrogenase (WblA protein)	5569740	5571050	1311	3	79	1.29	2.40E-02	COG0677	M	Cell wall/membrane/envelope biogenesis	CELLULAR PROCESSES AND SIGNALING				
plu4797	CDS	wblB	Myo-inositol 2-dehydrogenase (WblB protein)	5571065	5572114	1050	2	133	1.33	1.84E-02	COG0673	R	General function prediction only	POORLY CHARACTERIZED				
plu4801	CDS	wblE	WblE protein	5575036	5576142	1107	1	6	3.05	3.95E-04	COG5017	G	Carbohydrate transport and metabolism	METABOLISM				
pluCDS16291	CDS	-	-	1629184	1629867	684	-1	165	-1.58	3.51E-04	Unknow							
pluCDS22408	CDS	glbE	Similar to protein encoded in the glidobactin A synthetase gene	2240665	2240847	183	1	40	-1.8	1.55E-03	COG3039	X	Mobilome: prophages, transposons					
pluCDS25560	CDS	-	-	2556076	2556432	357	-1	558	-1.37	1.51E-03	COG3905	K	Transcription	INFORMATION STORAGE AND PROCESSING				
plutRNA5502	tRNA	-	transfert RNA-Thr	5502009	5502084	76	-1	8241	1.3	7.37E-03								

References

Duchaud, E., Rusniok, C., Frangeul, L., Buchrieser, C., Givaudan, A., Taourit, S., Bocs, S., Boursaux-Eude, C., Chandler, M., Charles, J.F., Dassa, E., Derose, R., Derzelle, S., Freyssinet, G., Gaudriault, S., Medigue, C., Lanois, A., Powell, K., Siguier, P., Vincent, R., Wingate, V., Zouine, M., Glaser, P., Boemare, N., Danchin, A., and Kunst, F. (2003
The genome sequence of the entomopathogenic bacterium Photorhabdus luminescens. Nat Biotechnol 21, 1307-131

Nielsen-Leroux, C., Gaudriault, S., Ramarao, N., Lereclus, D., and Givaudan, A. (2012).
How the insect pathogen bacteria Bacillus thuringiensis and Xenorhabdus/Photorhabdus occupy their hosts. Curr Opin Microbiol 15, 220-23
