# Supplementary material for: DNA Adenine Methyltransferase (Dam) Overexpression Impairs Photorhabdus luminescens Motility and Virulence
Source: Front Microbiol. 2017 Sep 1;8:1671. doi: 10.3389/fmicb.2017.01671 (PMC5585154; doi:10.3389/fmicb.2017.01671)
Supplement: Supplementary file 3 [file Table3.PDF]

Supplementary Table S3

List of 49 flagellar genes found in P. luminescens TT01 strain,
and their differential expression level between the Dam-overexpressing strain and its control strain,
based on RNAseq data

*** ratio Dam+ strain/control strain (red, underexpressed in the Dam overexpressing strain)**

** Bold, p value<0.05

The putative operonic structure is indicated by blue arrows beside the genes' name based on data
from *E. coli* and *Salmonella* (Chilcott et al., 2000)

	plu	Gene name	log2 fold change*	adjusted pvalue (FDR)**
**Cluster I**	plu1847	flhD	**-1.05**	**0.04**
	plu1848	flhC	**-1.15**	**0.01**
	plu1849	motA	**-1.09**	**0.04**
	plu1850	motB	-0.82	0.12
	plu1851	cheA	-0.61	0.30
	plu1852	cheW	-0.55	0.33
	plu1853	cheD	**-1.80**	**0.00**
	plu1854	tap	**-1.11**	**0.02**
	plu1855	cheR	-0.68	0.34
	plu1856	cheB	-0.45	0.62
	plu1857	cheY	-0.66	0.27
	plu1858	cheZ	-0.60	0.33
**Cluster II**	plu1895	flhB	0.00	1.00
	plu1896	flhA	-0.27	0.67
**Cluster III**	plu1912	flgN	-0.87	0.09
	plu1913	flgM	**-1.11**	**0.02**
	plu1914	flgA	-0.46	0.53
	plu1915	flgB	**-0.91**	**0.03**
	plu1916	flgC	-0.59	0.20
	plu1917	flgD	-0.57	0.22
	plu1918	flgE	-0.63	0.16
	plu1919	flgF	-0.47	0.34
	plu1920	flgG	-0.75	0.11
	plu1921	flgH	-0.13	0.91
	plu1922	flgI	-0.14	0.88
	plu1923	flgJ	-0.05	0.97
	plu1924	flgK	**-1.45**	**0.00**
	plu1925	flgL	**-1.63**	**0.00**
**Cluster IV**	plu1936	fliR	-0.32	0.63
	plu1937	fliQ	-0.47	0.51
	plu1938	fliP	-0.25	0.65
	plu1939	fliO	-0.34	0.51
	plu1940	fliN	-0.49	0.29
	plu1941	fliM	-0.58	0.18
	plu1942	fliL	-0.58	0.16
	plu1943	fliK	-0.37	0.50
	plu1944	fliJ	-0.23	0.82
	plu1945	fliI	-0.06	0.91
	plu1946	fliH	-0.29	0.62
	plu1947	fliG	-0.36	0.46
	plu1948	fliF	-0.44	0.33
	plu1949	fliE	-0.30	0.63
	plu1951	fliT	**-1.23**	**0.02**
	plu1952	fliS	**-1.21**	**0.02**
	plu1953	fliD	**-2.08**	**0.00**
	plu1954	fliC	**-3.35**	**0.00**
	plu1955	fliA	-0.38	0.66
	plu1956	fliZ	-0.51	0.30

Reference

Chilcott, G. S., and K. T. Hughes. 2000.
Coupling of flagellar gene expression to flagellar assembly in *Salmonella enterica* serovar typhimurium
and *Escherichia coli*. Microbiol Mol Biol Rev 64:694-708
