# Supplementary material for: DNA Adenine Methyltransferase (Dam) Overexpression Impairs Photorhabdus luminescens Motility and Virulence
Source: Front Microbiol. 2017 Sep 1;8:1671. doi: 10.3389/fmicb.2017.01671 (PMC5585154; doi:10.3389/fmicb.2017.01671)
Supplement: Supplementary file 4 [file Table4.PDF]

Supplementary Table S4

P. luminescens genes putatively involved in regulation of the chromosome replication, in mismatch repair (MMR), in double strand breaks (DSBs) repair and/or required for viability of *dam* mutants in other bacterial species. None of them showed a significant difference in expression in the *P. luminescens dam*-overexpressing strain.

Label	Begin	End	Length	Frame	Gene	Product	log2FoldChange	padj
plu0001	234	1622	1389	3	**dnaA**	Chromosomal replication initiator protein DnaA	-0.0518207589055677	0.997680032068507
plu0002	1627	2727	1101	1	**dnaN**	DNA polymerase III, beta chain	0.846304397460403	0.201662965393312
plu0003	2749	3840	1092	1	**recF**	DNA replication and repair protein	0.0846176574906654	0.970697136537198
plu0004	3860	6274	2415	2	**gyrB**	DNA gyrase subunit B	-0.0697211500242034	0.997680032068507
plu0005	6415	6894	480	1	**_**	Similar to Hcp protein	-0.0658956468439219	0.969630004815419
plu0050	44870	45310	441	-3	**mioC**	Protein mioC, involved in modulation of initiation at oriC, initiation of chromosome replication	-0.189801532644711	0.927841228043529
plu0339	362659	363132	474	1	**vsr**	Very short patch repair protein (DNA mismatch endonuclease) (Vsr mismatch endonuclease) (V.EcoKDcm)	-0.0100259517205973	0.995080928223759
plu0386	417018	419807	2790	3	**polA**	DNA polymerase I	0.083135162159989	0.968202378227714
plu0552	622889	624271	1383	2	**radA**	DNA repair protein RadA (sms)	0.18236546861866	0.923190120101124
plu0630	713763	717152	3390	3	**recC**	Exodeoxyribonuclease V gamma chain	-0.181579648744075	0.934203506186693
plu0632	720056	723664	3609	2	**recB**	Exodeoxyribonuclease V beta chain	0.0515759450379597	0.977003021015846
plu0722	830921	833476	2556	-3	**mutS**	DNA mismatch repair protein MutS	-0.113316130588219	0.977029176398937
plu1169	1353493	1354530	1038	-1	**mutY**	A/G-specific adenine glycosylase	-0.152872592493022	0.950845828359512
plu1249	1444202	1445269	1068	2	**recA**	RecA protein (Recombinase A)	-0.132698828293765	0.971766894615316
plu1406	1688390	1688914	525	2	**seqA**	SeqA protein, negative modulator of initiation of replication	-0.140905687617962	0.949619215739893
plu1784	2128483	2128803	321	-1	**yccV**	Hemimethylated oriC DNA-binding protein; weak repressor of dnaA transcription	-1.15062174589762	0.051094815936547
plu2110	2493753	2494274	522	3	**ruvC**	crossover junction endodeoxyribonuclease (Holliday junction nuclease RuvC) (Holliday junction resolvase RuvC)	-0.338803074503579	0.918856365490837
plu2111	2494361	2494978	618	2	**ruvA**	Holliday junction DNA helicase	-0.320273206059374	0.852105764563787
plu2112	2494995	2496002	1008	3	**ruvB**	Holliday junction DNA helicase	-0.425321467478446	0.866155280934776
plu2752	3263456	3264157	702	-3	**hda**	Required for regulatory inactivation of DnaA; multicopy suppressor of dnaN(ts) (by simialrity)	-0.319158123817258	0.891005488344538
plu2815	3341618	3345064	3447	2	**mfd**	transcription-repair coupling factor (TRCF)	-0.110958801845131	0.977029176398937
plu2859	3394549	3394968	420	1	**_**	putative Mut family protein	-0.135463956599561	0.950845828359512
plu3338	3965785	3966510	726	-1	**recO**	DNA repair protein recO (recombination protein O)	0.344909149968757	0.80653343217027
plu3374	4002348	4004009	1662	3	**recN**	DNA repair protein recN (recombination protein N)	-0.149412065969804	0.931683737733771
plu3610	4258106	4259008	903	2	**iciA**	Chromosome initiation inhibitor (OriC replication inhibitor)	-0.332414087454454	0.836539895258107
plu3644	4291033	4291425	393	-1	**mutT**	Mutator mutT protein (7,8-dihydro-8-oxoguanine-triphosphatase) (8-oxo-dGTPase) (dGTP pyrophosphohydrolase)	0.200449297044812	0.934339013099061
plu3678	4332988	4333674	687	1	**mutH**	DNA mismatch repair protein	-0.375401824183046	0.78847219117069
plu4344	5073287	5076445	3159	-3	**_**	putative NTPase involved in DNA repair	-0.0452502196468109	0.970697136537198
plu4374	5106205	5106810	606	-1	**lexA**	LexA repressor	-0.21631016426044	0.924379639012215
plu4583	5348451	5350346	1896	-2	**mutL**	DNA mismatch repair protein	0.167492620498627	0.917780401388868
plu4759	5540058	5542256	2199	3	**priA**	primosomal protein N' (replication factor Y)	0.0420304764865283	0.995080928223759
plu4857	5631647	5632456	810	-3	**mutM**	formamidopyrimidine-DNA glycosylase (FAPY-DNA glycosylase)	-0.368105439246214	0.791036525756647
plu4865	5639239	5639931	693	-1	**radC**	DNA repair protein	0.216422317083523	0.96788767205344
