# Supplementary material for: DNA Adenine Methyltransferase (Dam) Overexpression Impairs Photorhabdus luminescens Motility and Virulence
Source: Front Microbiol. 2017 Sep 1;8:1671. doi: 10.3389/fmicb.2017.01671 (PMC5585154; doi:10.3389/fmicb.2017.01671)
Supplement: Supplementary file 5 [file Presentation1.PDF]

Fig. S1

```
           1                                                50
     Plum  MKKKRAFLKW AGGKYPLVDD IKRHLPAGSR LIEPFVGAGS VFLNTDYDSY
     Xnem  MKKKRAFLKW AGGKYPLVDD IKRHLPEGDC LIEPFVGAGS VFLNTDYDSY
    Ecoli  MKKNRAFLKW AGGKYPLLDD IKRHLPKGEC LVEPFVGAGS VFLNTDFSRY
     Ahyd  MKKTRAFLKW AGGKYSLVEE IAERLPAGRV LLEPFVGAGS VFLNTDYDAY
       T4  ...MLGAIAY TGNKQSLLPE LKSHFPKYNR FVDLFCGGLS VSLNVNGPV.
Consensus  mkk.raflkw aGgKysLl.# ik.hlPkg.r lv#pFvGagS VfLNt#...y

           51                                               100
     Plum  ILADINSDLI NLYNTVKLRA DEFIVHTRPL FTQEYNTAEH FYQLREQFNQ
     Xnem  ILSDINSDLI NLYNTVKSRA DEFINHARPL FFPEFNTSEN FYRMREEFNK
    Ecoli  ILADINSDLI SLYNIVKMRT DEYVQAAREL FVPETNCAEV YYQFREEFNK
     Ahyd  VLNDINPDLI GLYNHLKRTP DSFIAEARKL FVAEHNHKAA YYRLRTQFNQ
       T4  ...EMYKRLI NVSWDDVLKV IKQYKLSKTS KEEFLKLRED YNKLANDIQE
Consensus  .l.#in.dLI nlyn..kl.. d......r.l f..e.n..e. %y.lr.#f#.

           101                                              150
     Plum  STDPFHRSVL FLYLNRHCYN GLCRYNSHGK FNVPFGRYKK PYFPEEELHW
     Xnem  SSDPFYRSIL FLYLNRHCYN GLCRYNSRGQ FNVPFGRYKK PYFPENELYW
    Ecoli  SQDPFRRAVL FLYLNRYGYN GLCRYNLRGE FNVPFGRYKK PYFPEAELYH
     Ahyd  ADTSFERALL FLFLNRHGFN GLCRYNKKGG FNVPFGSYKK PYFPEKELWA
       T4  PIITRDPLLL YV.LHFHGFS NMIRINDKGN FTTPFGKRTI NKNSEKQYNH
Consensus  ....f.r.lL %l.Lnrhg%n g$cRyN.kG. FnvPFG.ykk pyfpEk#l.h

           151                                              200
     Plum  FAEKAQNATF ICEHYQHTLN EAHKDSVIYC DPPYAPLSAT ANFTAYHTNS
     Xnem  FAEKSQKATF ICQHYEIALN NAPKGAVVYC DPPYAPLSAT ANFTAYHTNN
    Ecoli  FAEKAQNAFF YCESYADSMA RADDASVVYC DPPYAPLSAT ANFTAYHTNS
     Ahyd  FAEKAQKATF ICESYADAIQ RAEEDWVIYC DPPYAPLSTT ASFTTYSAGG
       T4  SSLHFKDVKI L......... ...DGDFVYV DPPY..LITV ADYNKFWSED
Consensus  faek.q.a.f .c..y..... .a.dg.v!Yc DPPYapLstt A.%t.%....

           201                                              250
     Plum  FNIADQENLA NIAFKLSSER AIPVLISNHD TPMTRKWYYQ ASLHIVKARR
     Xnem  FNLLDQENLA QIAYHLSSQR GIPVLISNHD TPMTREWYHQ ASLYIVKARR
    Ecoli  FTLEQQAHLA EIAEGLV.ER HIPVLISNHD TMLTREWYQR AKLHVVKVRR
     Ahyd  FTLDDQAVLA RLARHTAARK GVPVLISNHD IELTRELYRG ARLDEILVKR
       T4  FKQNCDKIIF E......EKD LLNLLDSLND RGI..KFGQS NVLEHHGKEN
Consensus  F....#..la e.a....... ..pvLiSnhD ...trk.yq. a.L......r

           251                                   290
     Plum  TISRNILARS KVDELLALYR .......... ..........
     Xnem  TISRNILARS KVDELLALYC QK........ ..........
    Ecoli  SISSNGGTRK KVDELLALYK PGVVSPAKK. ..........
     Ahyd  TISRNGGTRN KVAELLALYP PGIEPEQGYY PSDAELAPLG
       T4  TLLKEWSKKY NVKHLNKKYV FNIYHSKEKN GTDEVYIFN.
Consensus  tis.#...r. kV.eLlalY. ..i.....k. ..d.......
```

Figure S1. Sequence alignment of selected Dam MTase orthologs.

Plum, *P. luminescens* TT01 (Uniprot database accession number: Q7NA58); Xnem, *Xenorhabdus nematophila* F1 (N1NIZ0); Ecoli, *Escherichia coli* K-12 (P0AEE8); Ahyd, *Aeromonas hydrophila* (Q4PLJ4); T4, bacteriophage T4 (P04392). Red and bold underlined, amino-acid involved in GATC sequence recognition on DNA; highlighted in green, DPPY motif which corresponds to the active pocket site of the enzyme; highlighted in grey, amino-acids which interact with S-AdoMet.
