# Supplementary material for: DNA Adenine Methyltransferase (Dam) Overexpression Impairs Photorhabdus luminescens Motility and Virulence
Source: Front Microbiol. 2017 Sep 1;8:1671. doi: 10.3389/fmicb.2017.01671 (PMC5585154; doi:10.3389/fmicb.2017.01671)
Supplement: Supplementary file 6 [file Presentation2.PDF]

Fig. S2

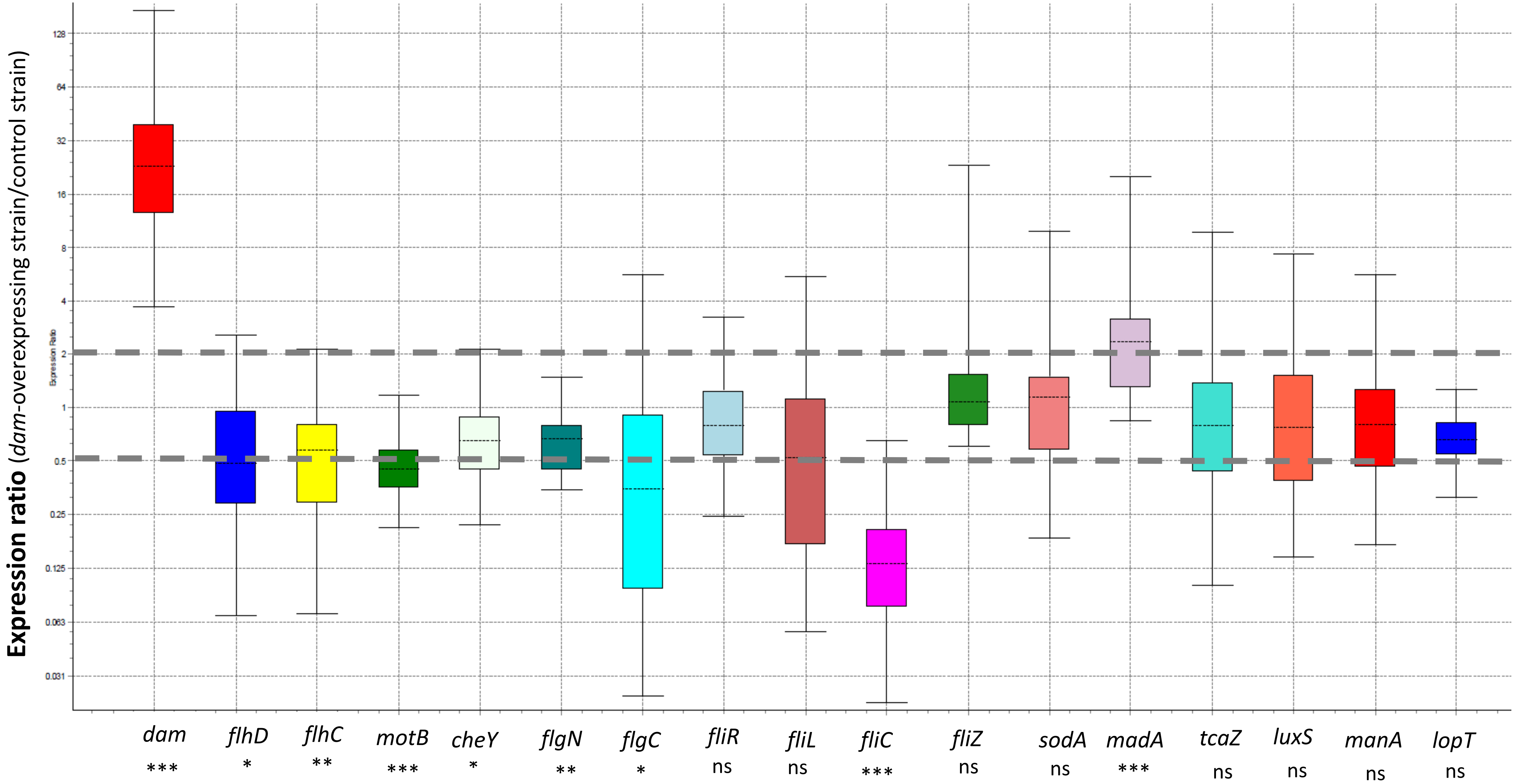

Figure S2. Relative expression of 17 genes in the *P. luminescens dam*-overexpressing strain.

qRT-PCR was carried out with total RNA extracted from exponentially growing cells of the *dam*-overexpressing strain and of the control strain. Box-plot representing the expression ratio between the two strains is represented for each tested gene, with *gyrB* used as a control gene (see Materials & Methods section for details). Expression ratios between 0.5 and 2 are flanked by doted gray lines. The level of expression between the 2 strains was different at $p<0.05$ (*), at $p<0.01$ (**), at $p<0.001$ (***), or was not significantly different (ns, $p>0.05$), depending on the tested genes.
